# Toxicokinetics of Arenobufagin and its Cardiotoxicity Mechanism Exploration Based on Lipidomics and Proteomics Approaches in Rats

**DOI:** 10.3389/fphar.2021.780016

**Published:** 2021-12-22

**Authors:** Lijuan Zhao, Lingyu Han, Xiaolu Wei, Yanyan Zhou, Yanqiong Zhang, Nan Si, Hongjie Wang, Jian Yang, Baolin Bian, Haiyu Zhao

**Affiliations:** ^1^ Key Laboratory of Beijing for Identification and Safety Evaluation of Chinese Medicine, Institute of Chinese Materia Medica, China Academy of Chinese Medical Sciences, Beijing, China; ^2^ Shaanxi Chinese Medicine Institute (Shaanxi Pharmaceutical Information Center), Xianyang, China; ^3^ School of Pharmaceutical Sciences, Tsinghua University, Beijing, China

**Keywords:** arenobufagin, cardiotoxicity, toxicokinetic, lipidomics, proteomics

## Abstract

Arenobufagin (ArBu), one of the main active bufadienolides of toad venom with cardiotonic effect, analgesic effect, and outstanding anti-tumor potentiality, is also a potential cardiotoxic component. In the present study, the cardiac effect of ArBu and its underlying mechanism were explored by integrating data such as heart rates, toxicokinetics, myocardial enzyme and brain natriuretic peptide (BNP) activity, pathological sections, lipidomics and proteomics. Under different doses, the cardiac effects turned out to be different. The oral dose of 60 mg/kg of ArBu sped up the heart rate. However, 120 mg/kg ArBu mainly reduced the heart rate. Over time, they all returned to normal, consisting of the trend of ArBu concentration-time curve. High concentrations of myocardial enzymes and BNP indicated that ArBu inhibited or impaired the cardiac function of rats. Pathological sections of hearts also showed that ArBu caused myocardial fiber disorder and rupture, in which the high-dose group was more serious. At the same time, serum and heart tissue lipidomics were used to explore the changes in body lipid metabolism under different doses. The data indicated a larger difference in the high-dose ArBu group. There were likewise many significant differences in the proteomics of the heart. Furthermore, a multi-layered network was used to integrate the above information to explore the potential mechanism. Finally, 4 proteins that were shown to be significantly and differentially expressed were validated by targeted proteomics using parallel reaction monitoring (PRM) analysis. Our findings indicated that ArBu behaved as a bidirectional regulation of the heart. The potential mechanism of cardiac action was revealed with the increased dose, which provided a useful reference for the safety of clinical application of ArBu.

## 1 Introduction

**FIGURE 1:**
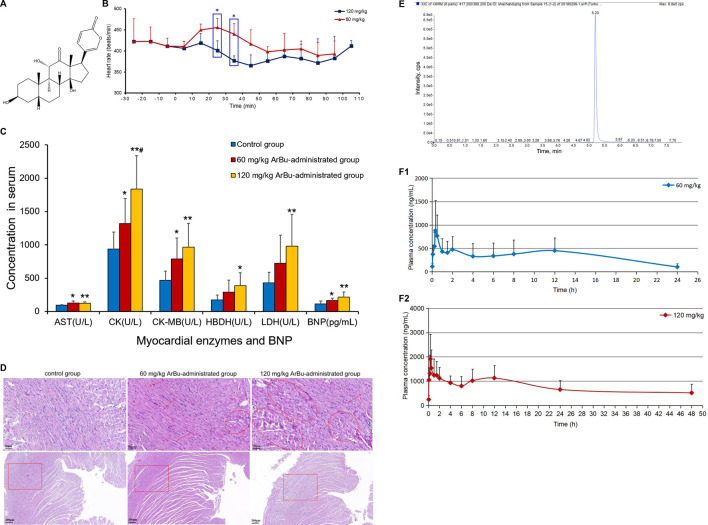
**(A)** Chemical structure of ArBu. **(B)** Heart rate diagrams in administration groups (*n* = 3), *means beats/min in the 60 mg/kg ArBu-administrated group was significantly different from that in 120 mg/kg ArBu-administrated group (*p < 0.05). **(C)** The myocardial enzyme and BNP levels in serum of three groups of rats (X ± SD, *n* = 8). compared with the control group, *p < 0.05, **p < 0.01, compared with the 60 mg/kg ArBu-administrated group, ^#^ *p* < 0.05. **(D)** Effect of ArBu on histopathological changes in heart tissues of mice. Heart tissues were taken after 2 h oral administration of vehicle or ArBu (*n* = 3). Four microscopic images were taken randomly from each sample; representative images were shown. The number of mice in each group is greater than or equal to 3. Scale bar = 50 µm **(up)**, 200 µm **(down)**. **(E)** The MRM chromatogram of ArBu in administrated plasma sample and its retention time was 5.2 min **(F-1)** The average concentration-time curves of 60 mg/kg ArBu-administrated group. **(F-2)** the average concentration-time curves of 120 mg/kg ArBu-administrated group. The data were expressed as mean value ±SD.

Arenobufagin (ArBu, C_24_H_32_O_6_) is a bufadienolide isolated from toad (*Bufo bufo gargarizans* Cantor and *Bufo melanostictus* Schneider) skin and toad venom (also called “Chan Su” in Chinese) (Figure 1A). In addition to the traditional cardiotonic and analgesic effects (Deng et al., 2015), ArBu is also a broad-spectrum anti-tumor active compound with notable effects on liver cancer, esophageal squamous cell carcinoma, non-small cell lung cancer (Zhang et al., 2013; Lv et al., 2017; Ma et al., 2017; Wei et al., 2020; Zhao et al., 2020). However, as a type B cardiac aglycone, excessive using of ArBu could cause arrhythmias, cardiac dysfunction and even death (Jiang et al., 2012; Kong et al., 2012). Research has demonstrated that the affinity of ArBu with the hearts might be stronger than other bufadienolides, such as bufalin, telocinobufagin, hellebrigenin, gamabufotalin, cinobufalin, bufotalin, resibufogenin and desacety-bufotalin (Jiang et al., 2011). It has been reported that the inhibitory effect of cardiac glycoside on Na^+^/K^+^- ATPase in cardiomyocytes is one of the main causes of arrhythmia (Qi et al., 2008). However, to our knowledge, there are still no reports about the integration of lipidomics and proteomics in the field of research on ArBu cardiotoxicity. As a potential anticancer drug candidate, the elucidation of its cardiotoxicity and mechanism will be of great significance.

**GRAPHICAL ABSTRACT:**
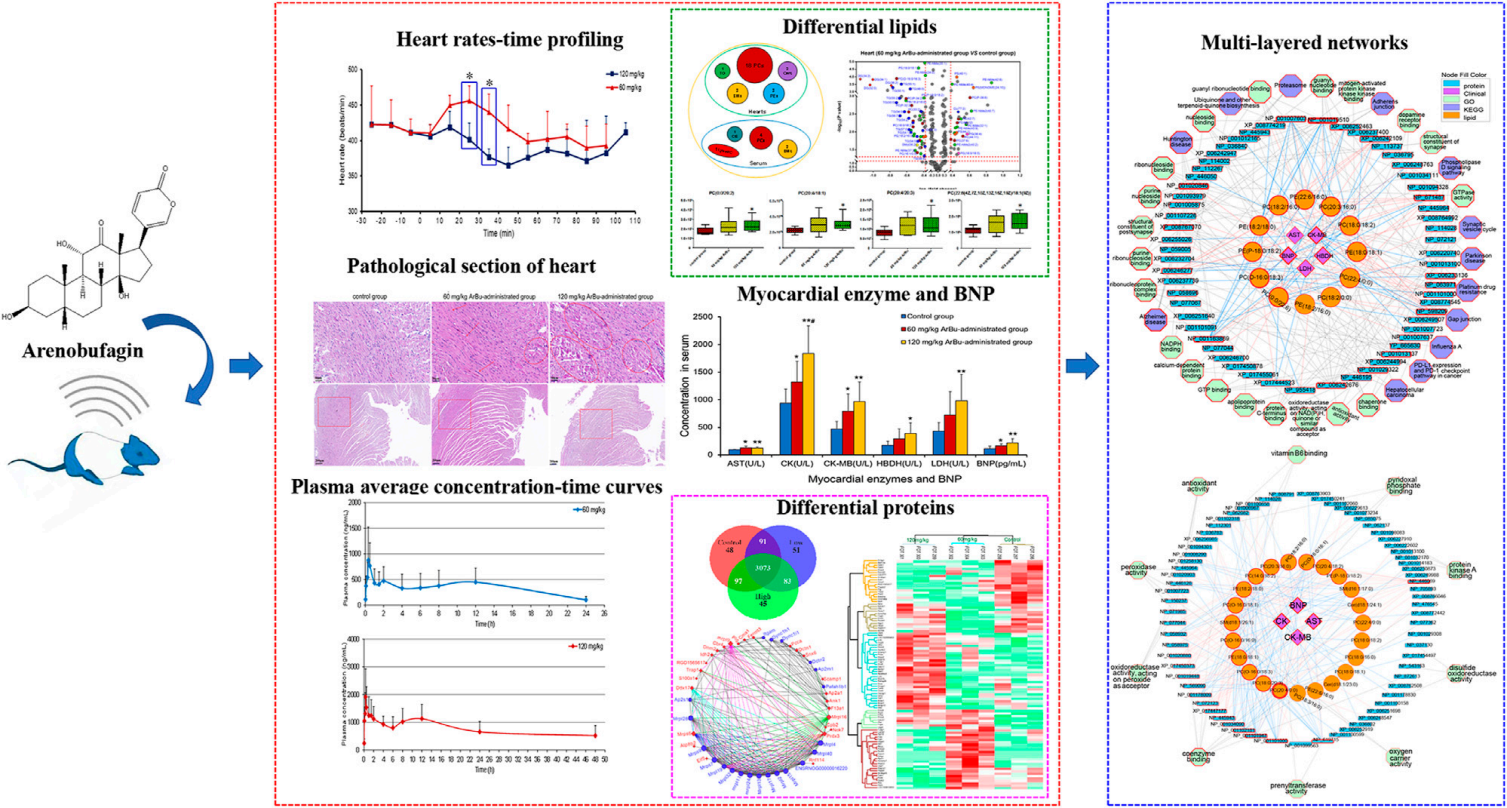


Lipids play an important part in cells. Under the action of phospholipase, they produce cell signaling factors and activate cellular signaling pathways, and are widely involved in and regulate various physiological activities of the human body (Watson, 2006; Bou et al., 2010). Research has indicated that toad venom may affect the normal heart function by interfering with lipid metabolism (Liang et al., 2010). As the major component in toad venom, the cardiotoxicity mechanism of ArBu might be closely relate to lipid metabolism. Multi-omics is a powerful tool to screen potential biomarkers, pathways and relations among differential substances (Dimitrakopoulos et al., 2018; Francescatto et al., 2018). Differential lipids and proteins could be combined to elucidate the cardiotoxicity mechanism of ArBu. Nowadays, advanced liquid chromatography-tandem mass spectrometry (LC-MS/MS) provides an important technical platform for lipidomics and proteomics research.

LC-MS/MS in parallel reaction monitoring (PRM) mode was used to verify the differential proteins. It was an antibody-independent targeted quantitative technology. Therefore, for some proteins without antibodies, and for proteins with post-translational modifications, quantitative results could be quickly obtained through PRM. The PRM mode had a high sensitivity and a good specificity. Its quantification range could reach more than 4 orders of magnitude, realizing accurate quantification of the protein in the true sense. In addition, in terms of throughput, an experiment using LC-MS/MS in PRM mode could simultaneously quantify up to dozens of proteins, which was also difficult for western blot to achieve.

Cardiotoxicity of ArBu was initially explored by determining the changes in heart rates, myocardial enzymes [including aspartate aminotransferase (AST), creatine kinase (CK), creatine kinase isoenzyme (CK-MB), lactate dehydrogenase (LDH) and hydroxybutyrate dehydrogenase (HBDH)], brain natriuretic peptide (BNP) and pathological sections. Furtherly, toxicokinetics, lipidomics and proteomics were being used to explore the cardiotoxic mechanism of ArBu. Oral administration of 60 mg/kg-ArBu accelerated heart rates in rats, while 120 mg/kg-ArBu initially accelerated and subsequently decreased the heart rates, consistent with the toxicokinetic results. They all returned to normal after 60 and 110 min, respectively. The levels of cardiac function assessment (CFA) indexes (myocardial enzymes and BNP) increased to various degrees, suggesting that ArBu might lead to inhibition or damage of cardiac function. Moreover, the pathological sections of hearts in high-dose (more serious) and low-dose ArBu groups showed certain cardiotoxicity (muscle fiber disorder or rupture). The major differentially-expressed lipids were cholesteryl ester (CE), phosphatidylcholines (PCs), sphingomyelins (SMs) in serum and PCs, phosphatidylethanolamines (PEs), triglyceride (TG) in hearts, respectively. The blood differential lipids in low-dose ArBu group was mainly related to BNP. While, the blood differential lipids in the high-dose ArBu group was associated with a variety of CFA indexes. Similarly, the differential proteins also showed significant differences among the groups. Multi-layered network analysis showed that the main pathways involved in the molecular mechanism of low-dose ArBu group were quite different from those of high-dose ArBu group. PRM verification showed that the regulation trends of ArBu on Trap1, Idh2, Mrps6 and Pcca were consistent with proteomics. In summary, high-dose ArBu caused inhibition or damage of cardiac function in rats. From cardiac excitation to cardiac inhibition, the underlying molecular mechanisms were quite different.

## 2 Materials and Methods

### 2.1 Chemicals

ArBu (No: 20150427, 97.2% purity) was purchased from Baoji Herbest Bio-Tech Co, Ltd. (Shanxi, China). The 0.9% sodium chloride solution was purchased from Hebei Tiancheng Pharmaceutical Co., Ltd. (Hebei, China). Chloral hydrate, propylene glycol and heparin sodium were purchased from Sinopharm Chemical Reagent Co., Ltd. (Shanghai, China). Ammonium acetate was purchased from Sigma-Aldrich (St. Louis, MO, United States). Thermo Scientific (United States) provided MS-grade methanol, formic acid, acetonitrile and protease inhibitor (Pierce) as well as trypsin inhibitor.

### 2.2 Instruments

The equipment and manufacturers used in the study were as follows: EMKA non-invasive electrocardiography telemetry system (Beijing GYD Labtech Co., Ltd., China), AU480 automatic biochemical analyzer (Olympus Corporation, Japan), DNM-9602G enzyme micro-plate reader (Beijing Perlong, China), DNX-9620A computer board washer, ABS2TM automatic blood collection instrument and ABS2 software (Instech Laboratories, Inc, United States), Mix-3000 shock mixer (Hangzhou Mio Instrument Co., Ltd., China), Mikro 220R high-speed refrigerated centrifuge (Hettich Scientific Instruments, Germany), and vacuum centrifugal concentrator (Thermo Scientific, United States).

### 2.3 Animals and Experimental Design

The animal studies were approved by the Animal Experimentation Committee of the Institute of Chinese Materia Medica, and were in compliance with the Nation Institute of Health guidelines for the Care and Use of Laboratory Animals. Specific pathogen free male Wistar rats (300 ± 30 g) were obtained from Beijing Vital River Laboratory Animal Technology Co., Ltd. (ethical review number SCXK (Beijing) 2016-0006). During the study, rats were exposed to suitable temperature (20°C–24°C) and humidity (40% RH–60% RH). The standard chow and tap water was adlibitum. They all fasted 12 h (h) before the experiment and had free access to water. Rats were anesthetized by intraperitoneal injection of 10% chloral hydrate (No: 20130201, Sinopharm Chemical Reagent Co., Ltd.) solution. Rats were randomly divided into three groups: a control group (propanediol + sodium chloride solution), a 60 mg/kg ArBu-administrated group and a 120 mg/kg ArBu-administrated group. ArBu was dissolved in propanediol with sodium chloride solution. The gavage volume was 2 ml.

### 2.4 Preparation of ArBu Suspension, Stock Solutions, Calibration Standards and Quality Control Samples

ArBu suspension was freshly prepared for intragastric administration. Namely, ArBu was dissolved in propylene glycol and diluted with 0.9% sodium chloride solution. The stock solution of ArBu (1.000 mg/ml) for the toxicokinetic study was prepared by dissolving ArBu in methanol and stored at −20°C before analysis. The ArBu calibration curve was obtained by serial dilution of the highest concentration (20000 ng/ml) calibration standard to the lowest concentration (100 ng/ml) calibration standard. The internal standard diazepam was prepared with a concentration of 1 μg/ml, 10 μg/ml, 5,000 ng/ml and 1,000 ng/ml of ArBu solutions were dried with nitrogen and then blank blood was added. Eventually, 1,000 ng/ml, 500 ng/ml and 100 ng/ml of ArBu solutions were obtained as high concentration quality control (HQC), medium concentration quality control (MQC) and low concentration quality control (LQC), respectively.

### 2.5 Detection of Heart Rates, Myocardial Enzymes and BNP in Rats and Pathological Section Examination

The abdominal hair was shaved with the electrodes attached. Rats were administered intragastrally with and without ArBu suspension (60 mg/kg, 120 mg/kg and control groups, *n* = 8), randomly after acclimatization. Following 120 min (min) of surveillance, the rats were anaesthetized with chloral hydrate. Blood was collected from the abdominal aorta and centrifuged at 21 g for 15 min at 7°C to obtain serum. After the sacrifice of the rats, hearts and livers were removed, frozen in liquid nitrogen and stored at −80°C before analysis. Serum levels of BNP and myocardial enzymes were determined.

The serum levels AST, CK, CK-MB, LDH and HBDH were determined by the machine of automatic biochemical analyzer AU480 made by American Beckman. AST and CK-MB levels were tested using an ultraviolet-malate dehydrogenase method and immunosuppressive assay, respectively. The activity of CK, LDH and HBDH in the serum was determined through the rating method. All myocardial enzyme tests were conducted in strict accordance with the instructions. The AST, CK, CK-MB, LDH and HBDH kits were purchased from InTec PRODUCTS, INC. (Xiamen, China). The BNP level was detected according to the instruction of ELISA Kit (Beijing Deyi clinical laboratory, China). The main instruments used were Beijing Perlong DNM-9602G enzyme label analyzer and DNX-9620A computerized plate washer.

In order to explore the effect of ArBu on the organs and tissues of rats, the pathological sections for 2 h after oral administration (control group, low-dose ArBu group, high-dose ArBu group, *n* = 3, at random) were analyzed as follows. Hearts and livers were fixed with formaldehyde, dehydrated, embedded in paraffin, sectioned, dewaxed and hematoxylin-eosin stained, which were made into pathological sections and detected.

### 2.6 Toxicokinetic Study in Rats

#### 2.6.1 Collection and Pretreatment of Plasma

After a 12 h fast, the rats were randomly divided into three groups (6 rats in each group): a control group (a mixture of propanediol and sodium chloride solution), a low-dose ArBu group (ArBu suspension 60 mg/kg) and a high-dose ArBu group (ArBu suspension 120 mg/kg). They all received a normal diet 4 h later. Plasma samples were collected from the common carotid vein at the following time points: 0, 2, 5, 15, 20, 30 min, 1, 1.5, 2, 4, 6, 8, 12, 24 and 48 h. The parameters for collecting plasma were as follows: sample volume, 225 μL, sample withdrawal rate, 375 μL/min, drug infusion rate, 375 μL/min, keep vein open rate, 100 μL/h, model, low loss, sample collection chamber temperature, 4–6°C. Plasma samples were collected into heparin tubes, in which 5 µL of 100 U heparin solution was added and volatilized overnight. Samples were mixed with heparin and centrifuged (2,500 g, 15 min, 4°C). Then, 90 µL of supernatant was transferred to a 1.5 ml Eppendorf tube and vortexed for 10 s 270 µL of acetonitrile and 9 µL of 1 μg/ml internal standard were added. The sample was vortexed for 2 min and centrifuged at 100 g for 10 min. The supernatant was transferred to a clean Eppendorf tube, dried under a nitrogen dryer and stored at −80°C before analysis. The dried residue was reconstituted with 90 µL of methanol, vortexed and centrifuged for MS analysis.

#### 2.6.2 Chromatographic Conditions

Analyses were conducted on an Agilent 1290 HPLC (Agilent, United States) equipped with AB SCIEX QTRAP 6500 system (AB SCIEX, United States). An Agilent Extend-C_18_ column (150 mm × 4.6 mm, 3.5 µm) was used for the gradient elution of the chromatographic separation. The mobile phases were 0.1% formic acid in water (A) and acetonitrile (B). The gradient program used was as follows: 0–2 min, 20% B, 2–4 min, 20%–90% B, 4–4.5 min, 90% B, 4.5–8 min, balance to 20% B. The flow rate was 0.3 ml/min. The injection volume was 1 µL.

ArBu was determined by monitoring the product-precursor transition in multiple reaction monitoring (MRM) mode using the positive ion acquisition mode. Other optimized mass parameters were as follows: curtain gas, 20, ion spray voltage, 5500 V, ion source gas 1, 55, ion source gas 2, 55, source temperature, 555°C, DP value of ArBu and diazepam, 140 V, collision energy of ArBu, 35 V, collision energy of diazepam, 41 V. The optimized quantification transition ion pairs (*m/z*) ranged from 417.2 to 399.2 for ArBu at 5.26 min and 285.3 to 193 for diazepam at 6.44 min, respectively.

#### 2.6.3 Method Validation

Selectivity was assessed by running plasma blank, ArBu-administrated plasma and plasma blank added with ArBu. Then, potential interferences from endogenous compounds were investigated. The calibration curve consisted of 8 blank plasma samples with an ArBu concentration of 5, 10, 20, 50, 100, 200, 500 and 1,000 ng/ml. The preparation method was in agreement with the previous plasma sample preparation. The lower limit of quantification was the concentration of ArBu with a signal-to-noise ratio greater than 10. The detection limit was at least triple the response (a signal to noise ratio ≥3) compared to blank, which was calculated by serial dilutions of the lower limit of quantification. QC samples (including LQC, MQC and HQC, *n* = 3), exposed at room temperature for 0, 6 and 12 h, were used for the evaluation of the stability of ArBu in plasma. The samples were considered stable if the test values were within the acceptable limits of ±15% relative standard deviation (RSD, %) in comparison with freshly prepared quality control samples.

The LQC, MQC, HQC samples were determined 6 times in 1 day and six consecutive days to calculate the intraday and inter-day precision and accuracy. Precision was expressed in %RSD. Accuracy was calculated as percentage deviation of the mean from the true value. The accuracy data were accepted if the precision measured in %RSD was within ±15%. ArBu extraction recovery was measured with LQC, MQC and HQC (*n* = 6) by comparing the areas of extracted ArBu samples with those of the post-extraction spiked ArBu samples. The area of the extracted ArBu sample was compared with the area of the extracted ArBu sample by LQC, MQC and HQC (n = 6). Then, the extraction recovery rate of ArBu was determined. The matrix effect was determined with LQC, MQC and HQC, comparing peak areas of post-extracted ArBu samples with peak areas of pure samples (Rama Raju et al., 2015).

#### 2.6.4 Toxicokinetic Analysis

The toxicokinetics of ArBu in rats was investigated by intragastric administration at doses of 0, 60 and 120 mg/kg (*n* = 6). During the analysis process, QC samples along with study samples were processed and distributed among the study samples. WinNonlin (Version 6.3, Pharsight Corporation, Mountain View, United States) software and NCA model were used to calculate and analyze the data obtained, respectively.

### 2.7 Lipid Profiling Analysis of Serum and Hearts

The serum and hearts obtained from each group (*n* = 8) at 2 h post-dose were used for lipidomics analysis. The serum samples were thawed on ice for 45 min before the pretreatment. 100 µL of serum and 300 µL of methanol were put into a 1.5 ml Eppendorf tube and vortexed for 15 s to precipitate proteins. The suspension was centrifugated at 100 g for 10 min (4°C). Additionally, the heart was ground with 1 ml of methanol, mixed for 30 min, followed by centrifugation at 100 *g* for 10 min (4°C). The supernatant taken from serum and hearts was used for lipidomics analysis.

Lipids was separated at 45°C using an UPLC CSH C_18_ column (100 mm × 2.1 mm, 1.7 µm) (Waters™, United States) on UltiMate™ 3000 Rapid Separation LC system (Thermo Scientific, United States) with a flow rate of 300 μL/min. Mobile phase A consisted of 10 mM ammonium acetate and 0.1% formic acid in acetonitrile-water (4: 6, v/v). Mobile phase B consisted of 10 mM ammonium acetate and 0.1% formic acid in isopropanol-acetonitrile (1: 9, v/v). The elution gradient was as follows: 0–1 min, 20% B, 1–16 min, 20%–100% B, 16–18 min, 100% B, 18–19 min, 100%–20% B, 19–19.5 min, 20% B. The injection volume was 1 µL. A Thermo Scientific™ Q Exactive hybrid quadrupole-orbitrap MS equipped with a HESI-II probe was employed. The MS parameters were as follows: positive HESI-II spray voltages, 3.7 kV, negative HESI-II spray voltages, –3.5 kV, heated capillary temperature, 320°C, *m/z* range, 50–1500, source temperature, 400°C, sheath gas pressure, 30 psi, auxiliary gas pressure, 10 psi, desolation temperature, 300°C, collision gas, nitrogen, collision gas pressure, 1.5 mTorr, resolution, 70000.

### 2.8 Proteomic Analysis of Hearts

Consistent with lipidomics, the hearts used for proteomics analysis were obtained from rats intragastrically administered with or without ArBu suspension for 2 h (three groups, *n* = 3, at random).

#### 2.8.1 Protein Sample Preparation

Whole tissue proteins were extracted as follows: 0.1 g of pooled cardiac tissues were lysed with 400 μL urea lysis buffers (8 M urea, 100 mM tris-HCl, pH 8.0). A 4 μL protease inhibitor was added to protect proteins from degradation. Protein concentrations were measured using the Bradford method (Eppendorf Biospectrometer).

#### 2.8.2 Protein Trypsin Digestion

Whole tissue proteins (100 μg) were digested by a FASP procedure. The protein samples were supplemented with 1 M dithiothreitol to a final concentration of 5 mM and incubated for 30 min at 56°C. Then iodoacetamide was added to a final concentration of 20 mM, and incubated in the darkness at room temperature. After half an hour of incubation, samples were added at final concentrations of 5 mM of dithiothreitol and retained in darkness for an additional 15 min. Following these procedures, protein samples were loaded into 10 kD Microcon filtration devices (Millipore) and centrifuged at 12,000 g for 20 min and washed twice with Urea lysis buffer (8 M Urea, 100 mM Tris-HCl, pH 8.0), twice with 50 mM NH_4_HCO_3_. Subsequently, the samples were digested with trypsin at an enzyme-to-protein mass ratio of 1:25 overnight at 37°C. The peptides were extracted and dried (SpeedVac, Eppendorf).

#### 2.8.3 LC-MS/MS Method for Proteomics

Orbitrap Fusion LC-MS/MS analyses were conducted on an Easy-nLC 1000 liquid chromatography system (Thermo Fisher Scientific) coupled to an Orbitrap Fusion Lumos *via* a nano-electrospray ion source (Thermo Fisher Scientific). The peptide samples were dissolved with the loading buffer (5% methanol and 0.2% formic acid), and loaded onto a C_18_ trap column (360 μm i.d × 2 cm) at a maximum pressure 280 bar with 12 μL solvent A (0.1% formic acid in water). The peptides were separated on another C_18_ column (150 μm i.d × 30 cm, 1.9 μm, 120 Å, Dr. Maisch GmbH) with a series of adjusted linear gradients according to the hydrophobicity of fractions with a flow rate of 600 nL/min. A mobile phase was composed of solvent A (0.2% formic acid-water) and solvent B (0.2% formic acid-acetonitrile) with gradient elution (0–150 min, 5%–40% B). The MS analysis was performed in a data-dependent manner with full scans (*m/z* 300-1,400) acquired using an Orbitrap mass analyzer at a mass resolution of 120,000 at *m/z* 200. The highest speed data-dependent mode was selected for fragmentation at normalized collision energy of 32%. The fragment ions were then transferred into the ion trap analyzer with the automatic gain control AGC target at 5e^3^ and maximum injection time at 35 ms. Dynamically excluding previously acquired precursor ions was activated at 20 s.

### 2.9 Differential Protein Verification Based on Targeted LC-MS/MS Analysis in PRM Mode

The sample preparation method was consistent with proteomics analysis with three biological replicates. For LC-MS/MS analysis, peptides were separated by a 120 min gradient elution at a flow rate 0.300 μL/min with a Thermo-Dionex Ultimate 3000 HPLC system, which was directly interfaced with the Thermo Orbitrap Fusion mass spectrometer. The analytical column was a homemade fused silica capillary column (75 μm i.d, 150 mm length; Upchurch, Oak Harbor, WA) packed with C-18 resin (300 Å, 5 μm; Varian, Lexington, MA). Mobile phase A consisted of 0.1% formic acid, and mobile phase B consisted of 100% acetonitrile and 0.1% formic acid. The Orbitrap Fusion mass spectrometer was operated in PRM mode using Xcalibur 4.2 software. The MS scans with a resolution of 120,000 were collected using a mass range of 300 and 1 400 m*/z*. Targeted MS^2^ spectra were acquired at 15000 resolution within auto scan range, after HCD with 30% NCE, and using an automatic gain control target value of 5e^4^ charges, a max IT of 22 millisecond and an isolation window of 1.6 m*/z*.

### 2.10 Statistics Analysis and Data Presentation

#### 2.10.1 Targeted Lipidomics

Based on retention time, precise mass-to-charge ratio and fragmentation information, a targeted database containing 193 lipids [including 97 PCs, 27 PEs, 24 SMs and 45 ceramides (Cers)] was established. Skyline (http://skyline.gs.washington.edu/) was used to import the LC-MS raw data for peak matching and to create the analytical template. Once the data table was exported and normalized, a multivariate analysis was performed using SIMCA-P 14.0 software (Umetric, Umea, Sweden), including principal components analysis (PCA) and orthogonal projection to latent structures discriminate analysis (OPLS-DA). Differential lipids were screened based on *t*-test (*p* < 0.05), variable influence in the projection (VIP, VIP >1) and fold change (FC_max (maximum)_, FC_max_ > 1.30 or FC_min (minimum)_, FC_min_ < 0.77).

#### 2.10.2 Untargeted Lipidomics

Progenesis QI data analysis (Nonlinear Dynamics, Newcastle, United Kingdom) was used to process the LC-MS raw data. Deconvolution, alignment and peak preparation produced a list of mass-to-charge ratio and retention time pairs with peak intensities. QC data was analysed to ensure the reliability of the sample data. The coefficient of variation for all samples above 20% was removed and multivariate analysis was performed. The basis of differential lipid identification was a precise mass-to-charge ratio (error less than 0.01). The Lipid Metabolites and Pathways Strategy (LIPID MAPS) Consortium databases and the Human Metabolome Database (HMDB) were used to identify differential lipids. Metabolic pathways were analysed through the MetaboAnalyst website (https://www.metaboanalyst.ca/).

#### 2.10.3 Label-Free Protein Quantification Method Proteomics

The raw MS files were handled with MaxQuant software (version 1.5.3.30). The identification of MS/MS-based peptides was carried out using the Andromeda search engine in MaxQuant. Andromeda used a target-decoy approach to identify peptides and proteins at an FDR <1%. As a forward database, the *Rattus norvegicus*_ref protein database (update to 2017/05/20, 66858 proteins ID in total) was used, which was downloaded from the National Center for Biotechnology Information Search database. A reverse database for the decoy search was generated automatically in MaxQuant. Enzyme specificity was set to “Trypsin.” At least 7 amino acids were required for peptide identification. The default settings were used for variable and fixed modifications [variable modification: acetylation (Protein-N terminus) and oxidation methionine], fixed modification: carbamidomethylation. MaxQuant label-free quantification algorithm was used to quantitate MS signals. Protein intensities were represented in intensity-based absolute-protein-quantification (iBAQ). The iBAQ from each sample was transferred into a fraction of total protein iBAQ amount per experiment (FOT). In addition, the kyoto encyclopedia of genes and genomes (KEGG), universal protein (UniProt) and gene ontology consortium (GO) databases were applied to perform functional annotation and identification of the protein biological process.

#### 2.10.4 PRM Validation of Differential Proteins

Raw files and target protein panel were loaded into SpectroDive 10.3 and analyzed with the default settings. SpectroDive would determine the ideal extraction window and perform quantification automatically. Qvalue cutoff on precursor was applied 1%. Peptides were manually inspected to verify matching spectra and peak integration. The area under curve (AUC) intensities of all fragments per precursor were summed to calculate the endogenous peptide signals.

## 3 Results and Discussion

### 3.1 Detection of Heart Rates, Myocardial Enzymes and BNP and Pathological Section Analysis

Changes in the rats’ heart rates reflected the cardiotoxicity degree of ArBu. For traditional experiments, rat heart rate tests were essentially recorded and analyzed under anesthesia, which lacked objectivity and delayed the time to obtain test results. The EMKA non-invasive electrocardiography telemetry system used in the present study detected the heart rates of rats under awake and non-traumatic conditions. The data obtained were more similar to normal physiological indicators. Furthermore, this method avoided errors in electrophysiological parameters caused by bodily stress (Wang et al., 2014). Integrated literatures (Liang et al., 2010; Jiang et al., 2011) and the pre-test of ArBu in rats, the oral administration of 60 and 120 mg/kg of ArBu was determined at two doses for cardiotoxicity studies. In the preliminary experiment, rats were at a risk of suffocating death as a result of intragastric administration following anesthesia. Therefore, the EMKA non-invasive electrocardiography telemetry system was used to detect the heart rates of conscious rats to reflect the true influences. The functioning and processing of the results was simple. The detection results indicated that heart rates in different administrated groups varied differently over time. Between 20 and 40 min after administration, there was a statistical difference in the number of heart beats per min between the 60 mg/kg ArBu administration group and the 120 mg/kg ArBu administration group (*p* < 0.05). 60 mg/kg ArBu accelerated the heart rates, while 120 mg/kg ArBu slightly increased the heart rates in the beginning and then slowed them down. However, the heart rates for the two groups returned to normal after approximately 60 and 110 min, respectively (Figure 1B).

Changes in heart rates after ArBu’s intervention were consistent with the dual effect of toad venom. Namely, low doses of toad venom could excite the hearts (cardiotonic effect), but high doses of toad venom could inhibit the hearts (toxic effect) (Li et al., 2016). In the present study, the heart rates of rats in the ArBu administration groups recovered over time, which was in keeping with the situation of toad venom (Liang et al., 2010).

In serum, AST, CK and CK-MB in the ArBu group administrated at 60 mg/kg were 1.3-fold, 1.4-fold and 1.7-fold higher than in the control group separately. Meanwhile, levels of AST, CK, CK-MB, HBDH and LDH in the 120 mg/kg ArBu-administrated group were 1.3-fold, 2.0-fold, 2.1-fold, 2.2-fold and 2.3-fold higher than in the control group, respectively. Among them, levels of CK increased in ArBu-dependent manner with a significant statistical difference between groups (Figure 1C). Levels of BNP were also up-regulated by ArBu to 1.4-fold in the low-dose ArBu group and 1.9 times higher in the high-dose ArBu group than in the control group with statistically significant. However, the difference between two ArBu dose groups was not statistically meaningful (*p* = 0.097) (Figure 1C).

The results suggested that ArBu increased serum levels of myocardial enzymes. Myocardial enzymes belonged to cytoplasmic enzymes, mainly existing in myocardium, brain tissue and skeletal muscle. When the myocardium was damaged, impaired cellular integrity led to the release and activity changes of enzymes. Remarkably, CK-MB was a specific myocardial enzyme, primarily distributed in the cytoplasm of myocardial cells. It was a key enzyme for monitoring heart function. BNP was a sensitive indicator of early heart impairment. In summary, overexpressed myocardial enzymes and BNP indicated that ArBu inhibited and damaged of heart function in rats.

In order to further determine the toxicity of ArBu to the organs, the hearts and livers of rats in each group were studied by pathological sections. Pathological sections of livers showed that ArBu had no hepatotoxicity (data not shown), which would be helpful for the development of ArBu anti-tumor drugs. In accordance with the results of myocardial enzyme assay, the pathological sections of hearts in high- and low-dose ArBu groups showed certain cardiotoxicity. As shown in Figure 1D, the pathological sections of the hearts in different groups were presented and compared. In low-dose group, muscle fiber disorder was found in many parts (arrows), the gap between muscle fibers was enlarged in general (panes), and muscle fiber rupture was found in a few parts (rings). While, in high-dose group, muscle fiber rupture occurred in more parts, indicating that myocardial cell damage was more serious. These results, together with the changes of heart rates and the increase of myocardial enzyme and BNP levels, demonstrated the cardiotoxicity of ArBu, and laid a foundation for the study of cardiotoxicity mechanism based on lipidomics and proteomics.

### 3.2 Toxicokinetic Study in Rats

#### 3.2.1 Method Validation

Analysis of the blank plasma, the plasma administrated by ArBu and the blank plasma added with ArBu showed that the shape and chromatographic separation of the ArBu MRM peak were presented well (Figure 1E). No peak interferences were detected at the retention time of ArBu (5.20 min). Eight samples of different concentrations were freshly prepared for constructing calibration curves. The ratio of the ArBu peak area to the internal standard (Yi/Ys) was plotted against the concentration (X, ng/mL). The calibration curve showed good linearity (range from 20 ng/ml to 4,000 ng/ml) with correlation coefficients (r) 0.9998. The regression equation was Y = 3.60242e^−4^ X + 0.00122. The limit of detection (S/N = 3/1) and the limit of quantitation (S/N = 10/1) was 0.1 and 0.01 ng/ml, respectively. The RSDs for the stability experiment were less than 15% after 0, 6 and 12 h. It ensured that biomonitoring and toxicokinetic studies could be carried out without unforeseen stability issues. Meanwhile, the intraday and inter-day precision showed RSD values less than 15%. The accuracy values of the intraday and inter-day validation were 88.9–110.4% (n = 6). It indicated that the instrument was stable and the detection method was feasible. The mean extraction recoveries of LQC, MQC and LQC were 93.6, 86.7 and 90.3% (*n* = 6), respectively. The method showed good matrix effect (80.2–97.5%). The results of extraction recoveries and matrix effect demonstrated that the pretreatment method was reasonable and feasible.

#### 3.2.2 Toxicokinetic Analysis

**FIGURE 2:**
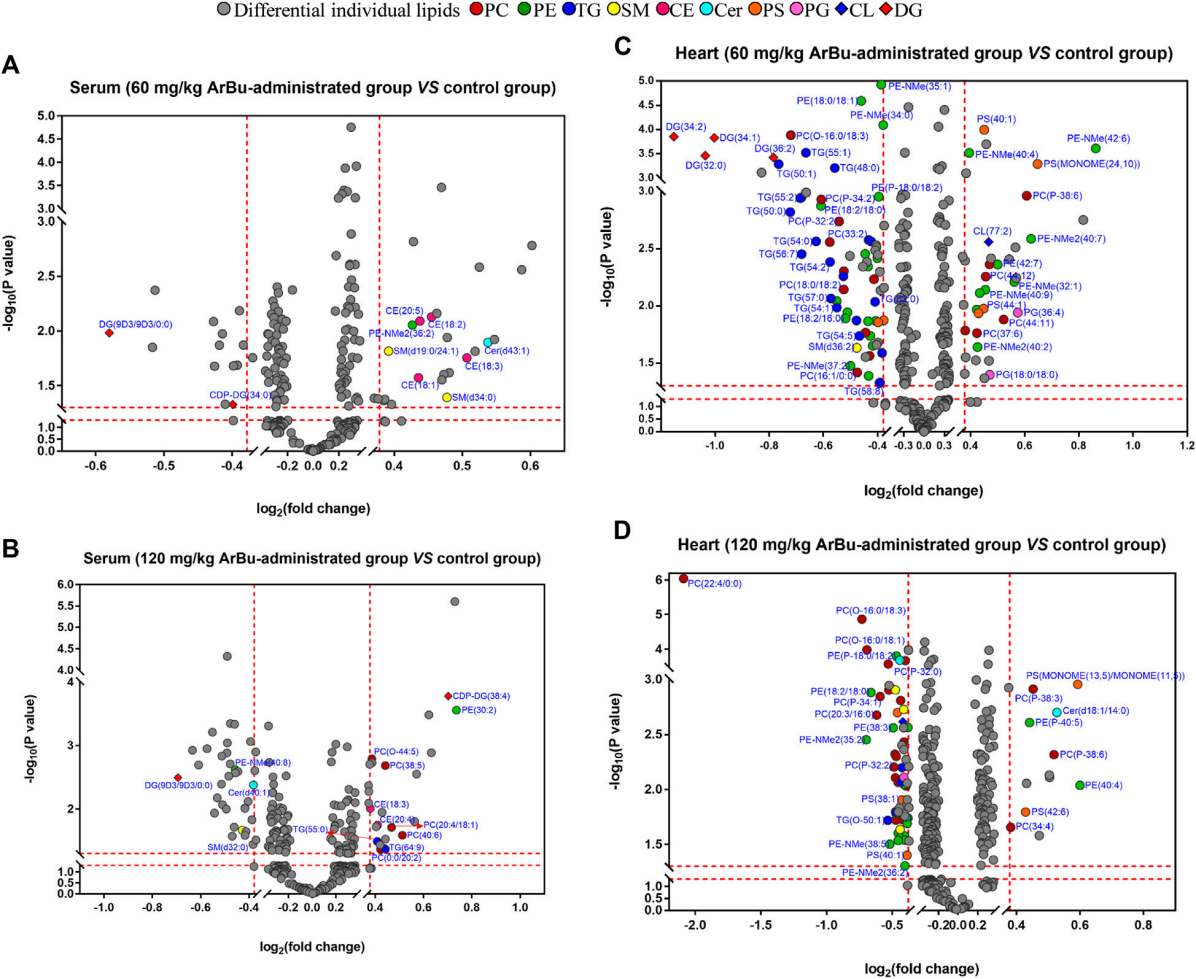
Volcano plot analysis of differentially-expressed lipids in hearts and serum. **(A)** Serum (60 mg/kg ArBu-administrated group vs. control group). **(B)** Serum (120 mg/kg ArBu-administrated group vs. control group). **(C)** Hearts (60 mg/kg ArBu-administrated group vs. control group). **(D)** Hearts (120 mg/kg ArBu-administrated group vs. control group).

Toxicokinetic samples collected from ArBu-treated rats at 0, 60 and 120 mg/kg were analyzed using the validated method. Toxicokinetic parameters were obtained by using the Winnonlin (Version 6.3, Pharsight Corporation, United States) software and the NCA model (Table 1). The mean blood concentration versus time profile of ArBu was shown in Figures 1F-1,1F-2. The time to maximum observed concentration of drug in plasma (C_max_) [time of C_max_ (T_max_)] of the 60 mg/kg ArBu- and 120 mg/kg ArBu-administrated groups were 0.61 and 0.64 h, respectively, which were very close. The C_max_ and mean residence time increased in an ArBu-dependent manner. As a result, apparent plasma clearance of the high-dose ArBu group was significantly lower than that of the low-dose ArBu group.

**TABLE 1:** Pharmacokinetic parameters of ArBu (n = 6, X ± SD).

Dose	HL_λ_z (h)	T_max_ (h)	C_max_ (ng/ml)	AUC_last_ (h*ng/mL)	CL/F (ml/h/kg)	MRT_last_ (h)
60 mg/kg ArBu	5.46 ± 2.03	0.61 ± 0.78	716.8 ± 280.6	9,089.2 ± 2,466.7	5,747.2 ± 1,448.1	9.12 ± 1.66
120 mg/kg ArBu	13.0 ± 2.69	0.64 ± 0.50	1863.2 ± 647.3	29606.3 ± 7,385.2	1,548.8 ± 859.8	17.89 ± 4.84

HL_λz_, elimination half-life; T_max_, time of C_max_; C_max_, maximum observed concentration of drug in plasma; AUC, area under the plasma concentration-time curve; CL/F, apparent plasma clearance; MRT, mean residence time.

The results demonstrated that ArBu entered the blood rapidly and was detected only after 2 min CYP3A4 was the major metabolic enzyme of bufogenins. Since bufalin was a strong inhibitor of CYP3A4 (Li et al., 2009), ArBu with the same parent nucleus was likely to inhibit CYP3A4. In combination with the results from the heart rate test, it was found that the rats’ heart rates in the 60 mg/kg ArBu-administrated group increased significantly at T_max_. However, in the 120 mg/kg ArBu-administrated group, the heart rates began to increase when the ArBu blood concentration increased to a certain amount. When it increased further, the heart rates became a decrease. With the rapid elimination of ArBu, heart rates returned to normal in ArBu administration groups when it dropped to a certain level. The time was 60 and 110 min for the low- and high-dose ArBu groups, respectively.

### 3.3 ArBu Regulated Distinct Lipid Profiles in Serum and Hearts of Rats

Serum and hearts from rats treated with ArBu for 2 h were obtained from each group (*n* = 8) for lipidomics research. The targeted lipidomics analysis was conducted using a targeted lipid database, including 97 PCs, 27 PEs, 24 SMs and 45 Cers. On the basis of accurate mass values and retention time, all the lipids were extracted and analyzed. In order to capture more potential biomarkers, an untargeted lipidomics method was combined with the targeted lipid analysis to systematically study the interference of ArBu on lipid metabolism. All the differentially-expressed lipids in serum and hearts were filtered out based on *p* < 0.05, VIP >1, and FC_max_ > 1.30 or FC_min_ < 0.77.

#### 3.3.1 Differential Lipids in Different Concentration

In serum, 13 differential lipids were found (FC_max_ > 1.30 or FC_min_ < 0.77) in the low-dose ArBu group, including CEs, Cer, diacyl glycerols (DGs), SMs, phosphatidylserines (PSs), PE and lyso-phosphatic acid (lyso-PA) (Figure 2A). However, 18 such differential lipids were identified in the high-dose ArBu group. They were PCs, PEs, TGs, DGs, CEs, SM, lyso-PA, cardiolipins (CL) and Cer (Figure 2B).

In hearts, a total of 83 differential lipids were obtained in the low-dose ArBu group compared with the control group. The subclasses of down-regulated lipids were mainly TGs (19/83), PEs (17/83), PCs (11/83), PSs (4/83), DGs (4/83) and PAs (2/83). While the subclasses of up-regulated lipids were mainly PEs (9/83), PCs (6/83), PSs (2/83), phosphatidylglycerols (PGs) (2/83) (Figure 2C). There were 70 differential lipids in the high-dose ArBu group compared with the control group. PCs (21/70), PEs (21/70), PSs (4/70), SMs (4/70) and TGs (3/70) were down-regulated, but PCs (5/70), PEs (2/70), PSs (2/70) and PAs (2/70) were up-regulated (Figure 2D).

#### 3.3.2 Differentially-Expressed Lipids in a Concentration Dependent Manner

**FIGURE 3:**
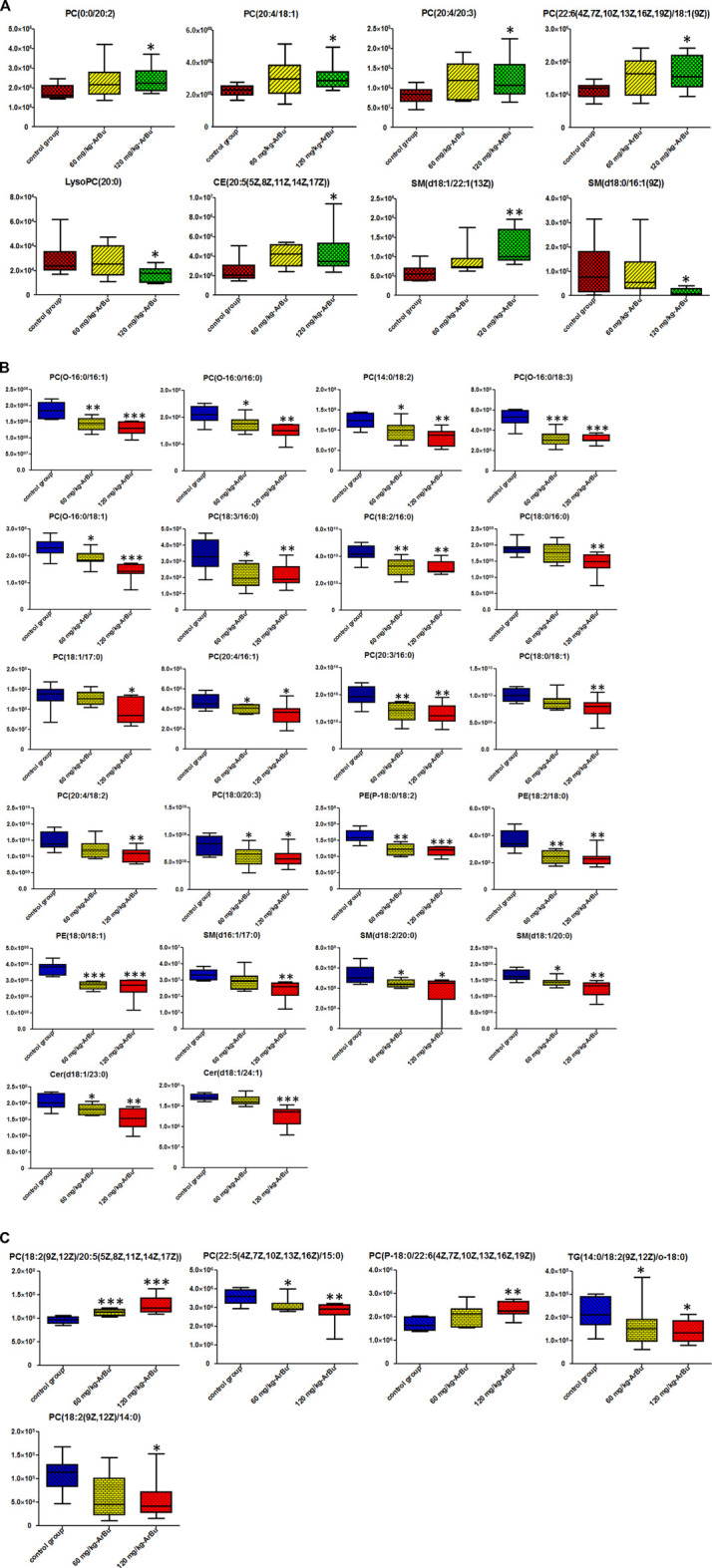
**(A)** Metabolic changes of multiple differential lipids in the serum of control group, 60 mg/kg ArBu- and 120 mg/kg ArBu-administrated groups of rats. **(B)** Metabolic changes of multiple targeted differential lipids in the hearts of control group, 60 mg/kg ArBu- and 120 mg/kg ArBu-administrated groups of rats. **(C)** Metabolic changes of multiple untargeted differential lipids in the hearts of control group, 60 mg/kg ArBu- and 120 mg/kg ArBu-administrated groups of rats. Box chart represent the normalized peak area. The data were expressed as mean value ±SD. *means differential lipids in the administration groups were significantly different from that in control group (*p < 0.05; **p < 0.01; ***p < 0.001).

In serum, eight differentially expressed lipids were discovered and identified in concentration dependent manners (Figure 3A; Table 2). Three potential lipid biomarkers, including PC(0:0/20:2), PC(20:4/18:1) and PC(20:4/20:3), were analyzed through a targeted lipidomics method. Five other potential lipid biomarkers (2 SMs, 1 CE, 1 PC and 1 lyso-PC) were found and identified by an untargeted lipidomics method. The results of the PCA and OPLS-DA score charts showed that the high-dose ArBu group separated clearly from the control group (Supplementary Figures S1A,B, S2A,C). Meanwhile, the *S*-plot loadings plot demonstrated the existence of differential lipids between the control and high dosage ArBu groups (Supplementary Figures S2B,D).

**TABLE 2:** Concentration dependent lipids in control, 60 mg/kg ArBu- and 120 mg/kg ArBu-administrated groups in serum (ESI^+^) of rats.

No	Potential biomarkers	t_R_/min	*m/z*	Formula	ID	VIP	Type	Max fold change (H/C)	C→L→H trend	Method
1	PC[0:0/20:2(11Z,14Z)]	7.72	548.3711	C_28_H_54_NO_7_P	—	—	PC	1.34	↑	targeted
2	PC[20:4(5Z,8Z,11Z,14Z)/18:1(9Z)]	9.15	808.5841	C_46_H_82_NO_8_P	LMGP01011908	—	PC	1.38	↑	targeted
3	PC[20:4(5Z,8Z,11Z,14Z)/20:3(8Z,11Z,14Z)]	9.03	832.5851	C_48_H_82_NO_8_P	LMGP01011918	—	PC	1.53	↑	targeted
4	PC[22:6(4Z,7Z,10Z,13Z,16Z,19Z)/18:1(9Z)]	10.78	876.5771	C_48_H_82_NO_8_P	HMDB08729	1.21	PC	1.41	↑	untargeted
5	LysoPC(20:0)	7.12	596.3942	C_28_H_58_NO_7_P	HMDB10390	1.23	LysoPC	1.75	↓	untargeted
6	CE[20:5(5Z,8Z,11Z,14Z,17Z)]	17.69	688.6029	C_47_H_74_O_2_	HMDB06731	1.81	CE	1.72	↑	untargeted
7	SM[d18:1/22:1(13Z)]	13.87	785.6527	C_45_H_89_N_2_O_6_P	HMDB12104	1.41	SM	2.09	↑	untargeted
8	SM[d18:0/16:1(9Z)]	12.03	747.567	C_39_H_79_N_2_O_6_P	HMDB13464	1.16	SM	7.10	↓	untargeted

P (H-C) < 0.05, VIP (H-C) > 1, FC_max_ (H/C) > 1.3, in an ArBu-dependent manner. C, control group; H, 120 mg/kg ArBu-administrated group.

In the hearts, 22 differential lipids were confirmed by the targeted lipidomics method, which included 14 PCs, 3 PEs, 3 SMs and 2 Cers (Figure 3B; Table 3). They all decreased in ArBu-dependent manners. Five differential lipids (4 PCs and 1 TG) (Figure 3C) were screened and identified after ArBu intervention in the untargeted lipidomics study (Table 3). PCA charts, OPLS-DA score charts and *S*-plot charts showed the presence of potential biomarkers between the high-dose ArBu group and the control group (Supplementary Figures S1C,D, S3). The main ArBu-dependent lipids expressed differentially were PCs and SMs in serum, while they were PCs, PEs and SMs in hearts. It could be concluded that ArBu regulated different types of lipids in serum and hearts, but PCs and SMs were co-affected.

**TABLE 3:** Concentration dependent lipids in control in the control, 60 mg/kg ArBu- and 120 mg/kg ArBu-administrated groups in hearts (ESI^+^) of rats.

No	Potential biomarkers	t_R_/min	*m/z*	Formula	ID	VIP	Type	Max fold change (H/C)	C→L→H trend	Method
1	PC[O-16:0/16:1(9Z)]	9.34	718.5745	C40H80NO7P	LMGP01020182	—	PC	1.44	↓	targeted
2	PC(O-16:0/16:0)	9.92	720.5902	C40H82NO7P	LMGP01020029	—	PC	1.43	↓	targeted
3	PC[14:0/18:2(9Z,12Z)]	8.49	730.5381	C40H76NO8P	LMGP01010496	—	PC	1.50	↓	targeted
4	PC[O-16:0/18:3(9Z,12Z,15Z)]	9.40	742.5745	C42H80NO7P	LMGP01020042	—	PC	1.66	↓	targeted
5	PC[O-16:0/18:1(9Z)]	10.02	746.6058	C42H84NO7P	LMGP01020003	—	PC	1.62	↓	targeted
6	PC[18:3(9Z,12Z,15Z)/16:0]	8.83	756.5538	C42H78NO8P	LMGP01011677	—	PC	1.61	↓	targeted
7	PC[18:2(9Z,12Z)/16:0]	9.11	758.5694	C42H80NO8P	LMGP01010932	—	PC	1.35	↓	targeted
8	PC(18:0/16:0)	10.20	762.6007	C42H84NO8P	LMGP01010742	—	PC	1.33	↓	targeted
9	PC[18:1(9Z)/17:0]	9.91	774.6007	C43H84NO8P	LMGP01011600	—	PC	1.38	↓	targeted
10	PC[20:4(5Z,8Z,11Z,14Z)/16:1(9Z)]	8.82	780.5538	C44H78NO8P	LMGP01011903	—	PC	1.34	↓	targeted
11	PC[20:3(8Z,11Z,14Z)/16:0]	9.20	784.5851	C44H82NO8P	LMGP01011872	—	PC	1.54	↓	targeted
12	PC[18:0/18:1(9Z)]	10.30	788.6164	C44H86NO8P	LMGP01010761	—	PC	1.31	↓	targeted
13	PC[20:4(5Z,8Z,11Z,14Z)/18:2(9Z,12Z)]	8.76	806.5694	C46H80NO8P	LMGP01011909	—	PC	1.39	↓	targeted
14	PC[18:0/20:3(8Z,11Z,14Z)]	9.97	812.6164	C46H86NO8P	LMGP01010799	—	PC	1.39	↓	targeted
15	PE[P-18:0/18:2(9Z,12Z)]	14.15	728.5589	C_41_H_78_NO_7_P	HMDB0011376	—	PE	1.38	↓	targeted
16	PE[18:2(9Z,12Z)/18:0]	10.68	744.5538	C_41_H_78_NO_8_P	HMDB0009090	—	PE	1.58	↓	targeted
17	PE[18:0/18:1(9Z)]	11.62	746.5694	C_41_H_80_NO_8_P	HMDB0008993	—	PE	1.48	↓	targeted
18	SM(d16:1/17:0)	10.56	689.5592	C_38_H_77_N_2_O_6_P	LMSP03010037	—	SM	1.39	↓	targeted
19	SM(d18:2/20:0)	9.55	757.6218	C_43_H_85_N_2_O_6_P	LMSP03010060	—	SM	1.43	↓	targeted
20	SM(d18:1/20:0)	10.16	759.6375	C_43_H_87_N_2_O_6_P	LMSP03010005	—	SM	1.33	↓	targeted
21	Cer(d18:1/23:0)	12.04	636.6289	C_41_H_81_NO_3_	LMSP02010021	—	Cer	1.34	↓	targeted
22	Cer[d18:1/24:1(15Z)]	11.75	648.6289	C_42_H_81_NO_3_	LMSP02010009	—	Cer	1.36	↓	targeted
23	PC[18:2(9Z,12Z)/20:5(5Z,8Z,11Z,14Z,17Z)]	11.03	804.5506	C_46_H_78_NO_8_P	HMDB08149	1.86	PC	1.34	↑	untargeted
24	PC[22:5(4Z,7Z,10Z,13Z,16Z)/15:0]	10.61	794.5690	C_45_H_80_NO_8_P	HMDB08658	1.45	PC	1.31	↓	untargeted
25	PC[P-18:0/22:6(4Z,7Z,10Z,13Z,16Z,19Z)]	11.82	818.6034	C_48_H_84_NO_7_P	HMDB11262	1.25	PC	1.37	↑	untargeted
26	PC[18:2(9Z,12Z)/14:0]	11.76	728.5242	C_40_H_76_NO_8_P	HMDB08130	1.37	PC	1.94	↓	untargeted
27	TG[14:0/18:2(9Z,12Z)/o-18:0]	17.95	834.7910	C_53_H_100_O_5_	HMDB42540	1.06	TG	1.57	↓	untargeted

P (H-C)<0.05, VIP (H-C) > 1, FC_max_ (H/C) > 1.3, in an ArBu-dependent manner. C, control group; H, 120 mg/kg ArBu-administrated group.

#### 3.3.3 Association of the Biological Significance of Differential Lipids With Cardiotoxicity

Biological meanings of differential lipids were analyzed based on the HMDB, KEGG databases and references. PCs were involved in the biosynthesis of phosphatidylcholine and glycerophospholipid metabolism. PCs with eicosapentaenoic acid chain captured our attention, such as PC[20:4(5Z,8Z,11Z,14Z)/18:1(9Z)], PC[20:4(5Z,8Z,11Z,14Z)/20:3(8Z,11Z,14Z)], PC [20:4(5Z,8Z,11Z,14Z)/16:1(9Z)] and PC [20:4(5Z,8Z,11Z,14Z)/18:2(9Z,12Z)]. PCs and PEs were the main sources of arachidonic acid (C20:4) in mammals. With respect to metabolism, arachidonic acid was mainly derived from PCs containing the eicosatetraenoic acid chain. PEs also served as a transient source which could be quickly replenished. Arachidonic acid was an important inflammatory mediator (Rouzer et al., 2006). Lyso-PC could inhibit glucose transport by binding to glucose transfer proteins on the cell membrane. It could also accelerate the hydrolysis of plasma membrane phospholipids by activating the PKC pathway, releasing more free arachidonic acid, thus promoting the inflammatory response (Yang et al., 2007). Furthermore, tyrosine phosphorylation would be inhibited when lyso-PC activated JNK or PKC, and cell structure and function would be abnormal when lyso-PC inhibited the activity of Na^+^-K^+^-ATPase (Zhao 2012). Lyso-PCs were involved in the metabolism of glycerophospholipids, for instance, lysoPC(20:0). As we know, lyso-PCs were the degradation products of phospholipids, which had high surface activity and could rupture red blood cells and other cell membranes, causing hemolysis or cell necrosis. The cardiotoxicity of ArBu might be associated with lysoPC(20:0). Furthermore, a study has been conducted on the effects of various lysophospholipids on cardiac sarcoplasmic reticulum calcium-transport activity in dogs. The results demonstrated that lysophospholipids decreased the activity of calcium transport. Moreover, lyso-PC was more effective than lyso-PS, lyso-PG and lyso-PE (Ambudkar et al., 1988). In this study, ArBu induced a dose-dependent decrease in lyso PC(20:0) in serum. Previous studies indicated that the imbalance of Ca^2+^ in cardiomyocytes was related to the cardiotoxicity of bufadienolide (Bick et al., 2002; Jiang et al., 2012; Li et al., 2014). Therefore, lysoPC(20:0) was a potential biomarker of the cardiotoxicity of ArBu. SMs participated in sphingomyelin metabolism and signal pathway. CEs were primarily involved in fatty acid and lipid metabolism.

PC[20:3(8Z,11Z,14Z)/16:0], PC[18:2(9Z,12Z)/20:5(5Z,8Z,11Z,14Z,17Z)], SM(d18:2/20:0), SM(d18:1/20:0) and lysoPC(20:0), as potential biomarkers, could be broken down into C20 fatty acids. C20 fatty acids could further synthesize important inflammatory mediators and signaling substances, such as prostaglandins, tranexamic acids, leukotrienes and peroxyarachidonic acids (Luo et al., 2011). These substances would cause metabolic disorders of the inflammatory mediators. Glycerophospholipids with 16: 0, 16: 1, 18: 0, or 18: 1 fatty acid accounted for 50% of serum markers and 75% of heart markers. These differential glycerophospholipids were involved in multiple metabolic pathways, such as the glycerophospholipid metabolism pathway, the arachidonic acid metabolism pathway, linoleic acid metabolism, α-linoleic acid metabolism, the body’s integrated metabolism and the retrograde endocrinology signaling pathway. The results of pathway analysis indicated that disorders of glycerophospholipid metabolism were likely to cause inflammatory responses (Zhao 2012). Interestingly, PE[18:0/18:1(9Z)], a potential biomarker in the heart, was probably the instantaneous source of arachidonic acid. Therefore, the level of PE[18:0/18:1(9Z)] decreased significantly when it was transformed into arachidonic acid. In addition, one third of the differential lipids in the heart contained a C18:2 chain. Linoleic acid from hydrolysis of these glycerophospholipids was the precursor of arachidonic acid. Meanwhile, linoleic acid was also the raw material for the metabolism of other inflammatory mediators. Increased linoleic acid could also contribute to the development of inflammatory by increasing the synthesis of C20: 4 and other fatty acids of the C20 family (Luo et al., 2011). The main affected pathway in serum and hearts was glycerophospholipid metabolism, which was a potential target for cardiotoxicity of ArBu (Supplementary Figure S4).

When the different dose ArBu group was compared with the control group respectively, for the hearts, the changes in PEs, PCs and PSs were significantly different. Remarkably, there were significant differences in TGs, DGs, PAs and SMs between high and low dose ArBu groups. Among the differential lipids in serum, Cer and SM were regulated upward in the low-dose ArBu group and regulated downward in the high-dose ArBu group. PSs was only found in the low-dose ArBu group. However, PCs, TGs and CL only existed in the high-dose ArBu group. In summary, the differential lipids in the hearts were significantly more numerous than in the serum. The major lipid subclasses were PCs, PEs and TGs in the hearts. Meanwhile, the differential lipids obtained from different dose ArBu groups were more different than the same in quantity and trend in both hearts and serum.

**FIGURE 4:**
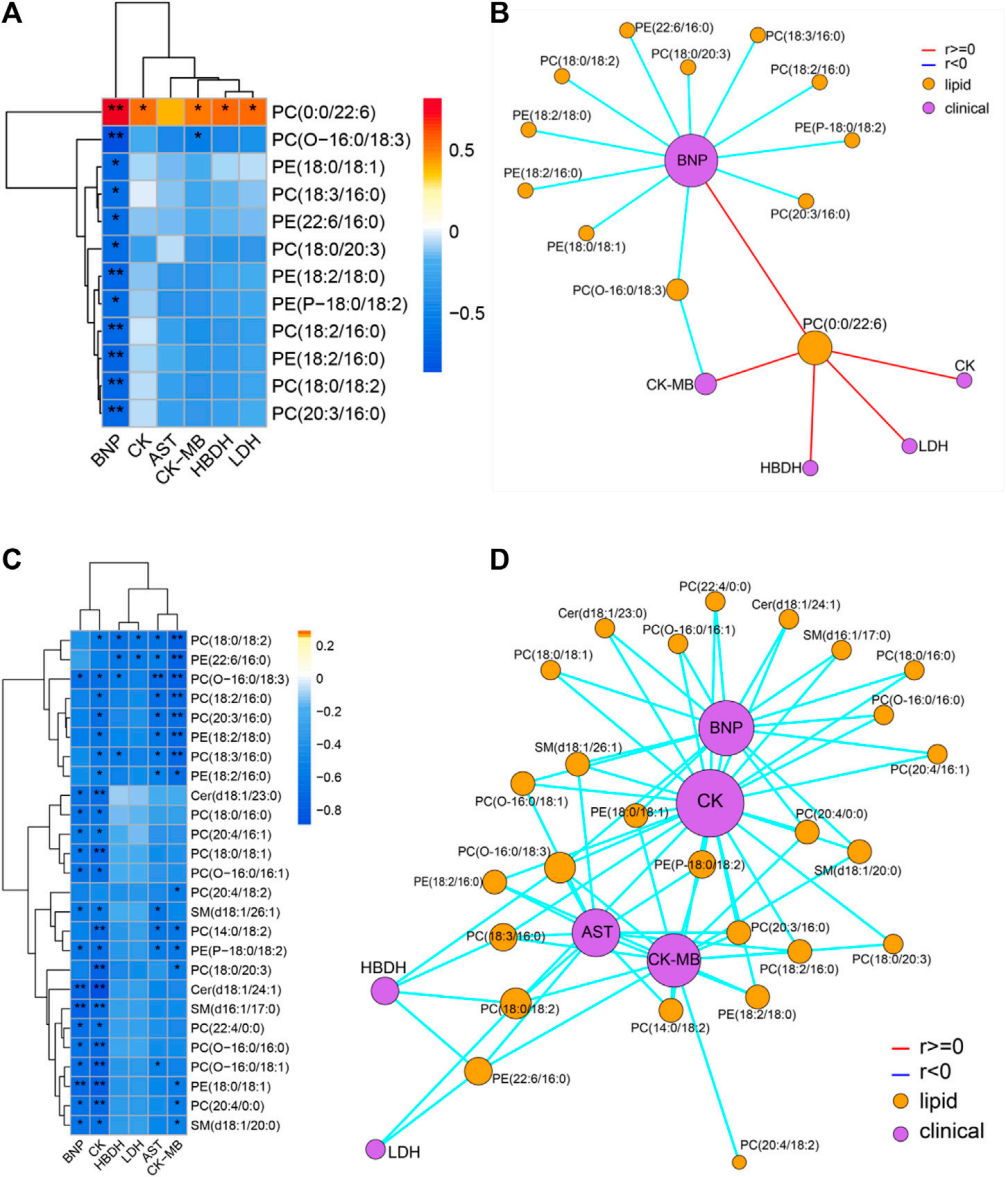
**(A)** Heatmap of lipids and CFA indexes based on spearman analysis (60 mg/kg ArBu-administrated group vs. control group). **(B)** Network of lipids and CFA indexes based on spearman analysis (60 mg/kg ArBu-administrated group vs. control group). **(C)** Heatmap of lipids and CFA indexes based on spearman analysis (120 mg/kg ArBu-administrated group vs. control group). **(D)** Network of lipids and CFA indexes based on spearman analysis (120 mg/kg ArBu-administrated group vs. control group). *means differential lipids were significantly different from CFA indexes (*p < 0.05; **p < 0.01).

Spearman correlation analysis was carried out the correlation between the targeted differential lipids of different ArBu dose groups with CFA indexes respectively (Figure 4). These differential lipids were all screened using *p* < 0.05 and an absolute value of r > 0.5. The results showed that only PC(0:0/22:6) was positively correlated with multiple CFA indexes among the 12 differential lipids in the low-dose ArBu group. The remaining 11 differential lipids all showed a negative correlation and only a significant correlation with BNP. However, these lipids were significantly correlated with multiple indexes of CFA in the high-dose ArBu group. In addition, there were 15 differential lipids in the high-dose ArBu group, negatively correlated with a number of CFA indexes, which could be used as potential biomarkers of ArBu cardiotoxicity. Overall, the differential lipids in the low-dose ArBu group were mainly related to BNP. However, they were CK > BNP = CK-MB > AST in the high-dose ArBu group. This was also an important basis for distinguishing the cardiotoxicity from ArBu.

### 3.4 ArBu Regulated Distinct Protein Profiles in Hearts of Rats

**FIGURE 5:**
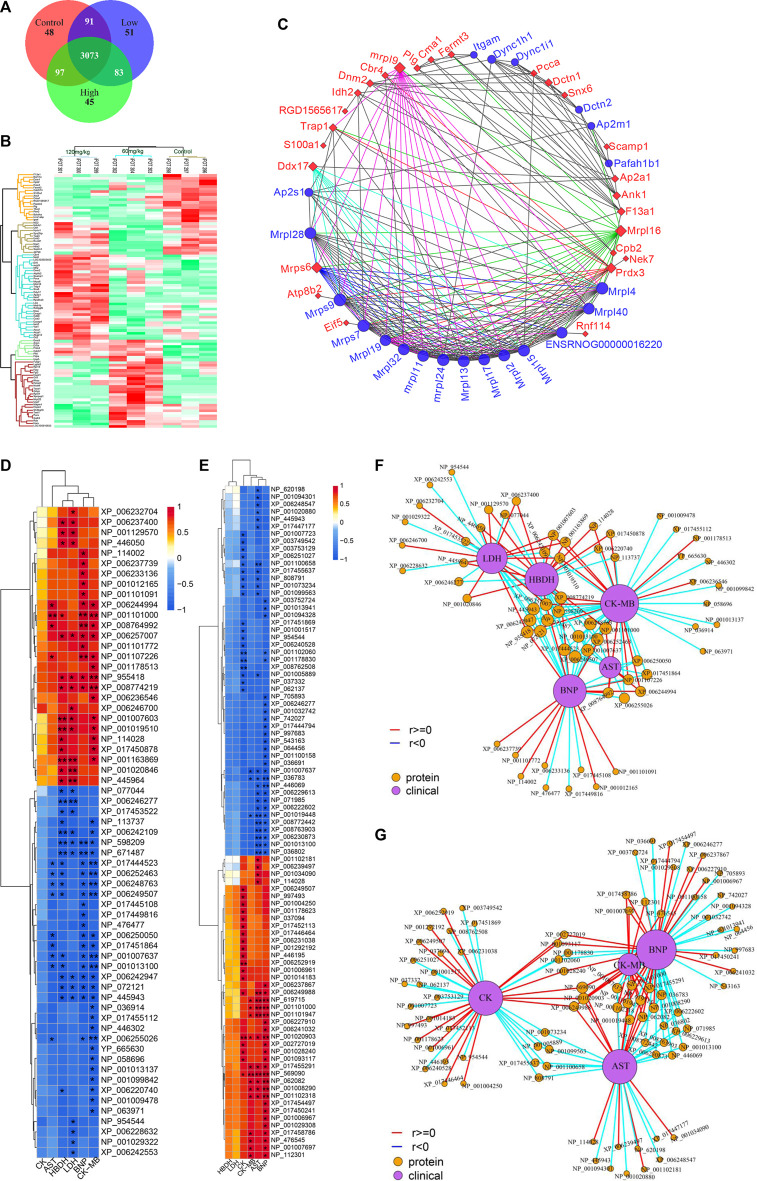
**(A)** Venn diagrams of detected proteins in the hearts of rats of control, low- and high-dose ArBu groups. **(B)** Thermogram analysis of differentially-expressed proteins in hearts. **(C)** PPI of differential proteins. **(D)** Heatmap of proteins and CFA indexes based on spearman analysis (60 mg/kg ArBu-administrated group *VS* control group). **(E)** Heatmap of proteins and CFA indexes based on spearman analysis (120 mg/kg ArBu-administrated group vs. control group). **(F)** Network of proteins and CFA indexes based on spearman analysis (60 mg/kg ArBu-administrated group vs. control group). **(G)** Network of proteins and CFA indexes based on spearman analysis (120 mg/kg ArBu-administrated group vs. control group). *means differential lipids were significantly different from CFA indexes (*p < 0.05; **p < 0.01).

The rat hearts used for proteomics study were also obtained 2 h after administration of ArBu. Proteomics was conducted by Orbitrap Fusion LC-MS/MS. A total of 3,488 proteins were detected (Figure 5A). Based on *p* < 0.05 and FC_max_ > 1.3 (or FC_min_ < 0.77), the differential proteins of the ArBu-administrated groups were screened. The results showed that 31 of 3,073 proteins were up-regulated and 47 of 91 proteins were down-regulated in the low-dose ArBu group. However, 49 of 3,073 proteins were up-regulated and 55 of 97 proteins were down-regulated in the high-dose ArBu group. When three groups were analysed simultaneously, 95 ArBu-dependent differential proteins were selected from 3,073 proteins (Figure 5B). Among them, 17 ArBu-dependent differential proteins were up-regulated and 12 ArBu-dependent differential proteins were down-regulated (Table 4). Then, the biological significance of these differential proteins were further analysed in the UniProt and Go databases. The downward-regulated proteins were mainly related to coagulation hemostasis and wound healing, while the upward-regulated proteins mostly located in mitochondria were associated with substance synthesis and energy metabolism.

**TABLE 4:** Differentially-expressed proteins in an ArBu-dependent manner in rat hearts.

No	Gene symbol	Description	C→L→H trend
1	F13a1	Coagulation factor XIII A chain	↓
2	Rnf114	E3 ubiquitin-protein ligase RNF114	↓
3	Dctn1	Dynactin subunit 1	↓
4	Cpb2	Carboxypeptidase B2	↓
5	Prdx3	Peroxiredoxin 3	↓
6	Fermt3	Fermitin family member 3	↓
7	S100a1	Protein S100-A1	↓
8	Ank1	Ankyrin 1	↓
9	Cma1	Chymase	↓
10	RGD1565617	—	↓
11	Popdc2	Popeye domain-containing 2	↓
12	Plg	Plasminogen	↓
13	Cbr4	Carbonyl reductase family member 4	↑
14	Nebl	Nebulette	↑
15	LOC102555453	—	↑
16	Eif5	Eukaryotic translation initiation factor 5	↑
17	mrpl9	39S ribosomal protein L9, mitochondrial	↑
18	Idh2	Isocitrate dehydrogenase (NADP), mitochondrial	↑
19	Dnm2	Dynamin-2	↑
20	Atp8b2	Phospholipid-transporting ATPase	↑
21	Scamp1	Secretory carrier-associated membrane protein 1	↑
22	Pcca	Propionyl-CoA carboxylase alpha chain, mitochondrial	↑
23	Mrps6	Mitochondrial ribosomal protein S6	↑
24	Mrpl16	39S ribosomal protein L16, mitochondrial	↑
25	Trap1	Heat shock protein 75 kDa, mitochondrial	↑
26	Snx6	—	↑
27	Ddx17	—	↑
28	Ap2a1	AP-2 complex subunit alpha	↑
29	Nek7	Serine/threonine-protein kinase Nek7	↑

The protein-protein interaction (PPI) network of differential proteins (Figure 5C) was analyzed using String website and Cytoscape software (Shannon et al., 2003). It could be seen from the PPI diagram that Mrpl16, Mrps6, Prdx3, F13a1, mrpl9 and Ddx17 were closely related to other proteins. Fermt3, down-regulated by ArBu, was related to blood coagulation and hemorrhagic diseases. Fermt3 level affected the function of platelet and inflammatory process (Oksala et al., 2015). ArBu also reduced the expression of Plg and CPB2. They were important node proteins in the protein-lipid association network. Both were connected with blood coagulation and inflammation. Plg was plasmin zymogen, which was very important to degrade fibrin clots. And it could interact with cell surfaces specifically (Miles et al., 2014). CPB2 had an important bearing on coagulation, thrombosis, inflammation and innate immunity (Leung and Morser, 2018). The blood coagulation factor XIIIA chain (F13a1) could be activated by thrombin and calcium ions to transglutaminase, which catalyzed the formation of γ-glutamyl-ε-lysine cross-links between fibrin chains to stabilize fibrin clots.

As we know, mitochondria are the most important energy supply site for cell life activities, providing about 95% of the energy of cells (Gunter et al., 2004). Meanwhile, mitochondria regulated the balance of calcium ion transport (Pizzo et al., 2012). The intracellular calcium concentration interacted with mitochondrial energy metabolism levels (McCormack and Denton, 1980; Cortassa et al., 2003). Mrps6, mrpl9 and Prdx3 were important nodal proteins in PPI. Mrps6 was also associated with cardiovascular disease, myocardial infarction and coronary artery disease. Trap1 was an important node protein in the protein-lipid association network and involved in regulating the cell cycle and mitochondrial respiration (Palladino et al., 2016).

In previous mechanism studies of cardiotoxicity for bufadienolide revealed that calcium ion disorder in cardiomyocytes was related to heart rate disorders (Bick et al., 2002; Luo et al., 2011; Jiang et al., 2012; Li et al., 2014), suggesting that the differential proteins might affect the change of calcium ion concentration. The difference was that S100A1 affected calcium-dependent regulation of cardiac contraction and relaxation, and the levels of myocardial enzymes and BNP. S100A1 played an important role in the inflammatory response caused by myocardial hypoxia. Meanwhile, it regulated the TLR4/ROS/NF-κB pathway to affect the oxidative stress and inflammatory response in H9C2 cells (Yu et al., 2015). Snx6 could inhibit the degradation of E-cadherin, which was related to cell signal transduction and Na, K-ATPase activity. CMA1 was associated with coronary heart disease. It might also be related to hypertension. CMA1 polymorphisms might be linked to atrial fibrillation (Zhou et al., 2019). Atp8b2 was also related to mitochondrial energy metabolism and lipids, such as flipping enzyme activity for PC and vesicle formation phospholipid transporters.

Some important differential proteins (Figures 5D–G) were also related to heart-related diseases and lipids. Up-regulated Golga4 was an important node in the protein-lipid association network and a potential biomarker of cardiac hypertrophy. Down-regulated Xirp2 was also an important node in the protein-lipid association network. It was related to cardiotoxicity, heart remodeling, cardiac arrhythmia diseases, early onset of heart failure, hypertrophic cardiomyopathy and cardiovascular diseases. Up-regulated Ddx17 was an important node in PPI, which was related to cardiac injury, mitochondrial energy metabolism and atherosclerosis. The concern is that, the relationship between phospholipids and coagulation was extremely close. As an anionic phospholipid, PS was often located on the inner side of the cell membrane (Yeung et al., 2008). PS exposure in red blood cells, platelets and endothelial cells increased thrombin production (Dong et al., 2013). As a cofactor in hemostasis and thrombosis, PS caused thrombin by supplying a catalytic surface for FXa and prothrombinase complexes (Kay and Grinstein, 2013). PS-exposing blood cells played an important role in hypercoagulability in non-valvular atrial fibrillation patients (Wang et al., 2018). As a calcium-dependent lipid scramblase, TMEM16F mediated PS exposure on the plasma membrane. Meanwhile, TMEM16F could regulate platelet function (Brooks et al., 2015). In addition, there were some important difference proteins. Rnf114 was involved in the regulation of NF-κB activity to help control T cell-mediated immune response (Rodriguez et al., 2014). Popdc2 in mice and zebrafish showed an overlapping effect in electrical conduction in the heart (Brand et al., 2014). Nek7 acted as a mutually exclusive cell switch for mandatory inflammatory response and cell division (Shi et al., 2015).

The conclusion of the above literature search, heatmap and network of proteins and CFA indexes based on spearman analysis was that important differential proteins were closely linked to blood coagulation, calcium ions, mitochondrial energy metabolism, lipids and heart-related diseases.

### 3.5 Potential ArBu Cardiotoxicity Mechanism Based on Multi-Layered Networks

**FIGURE 6:**
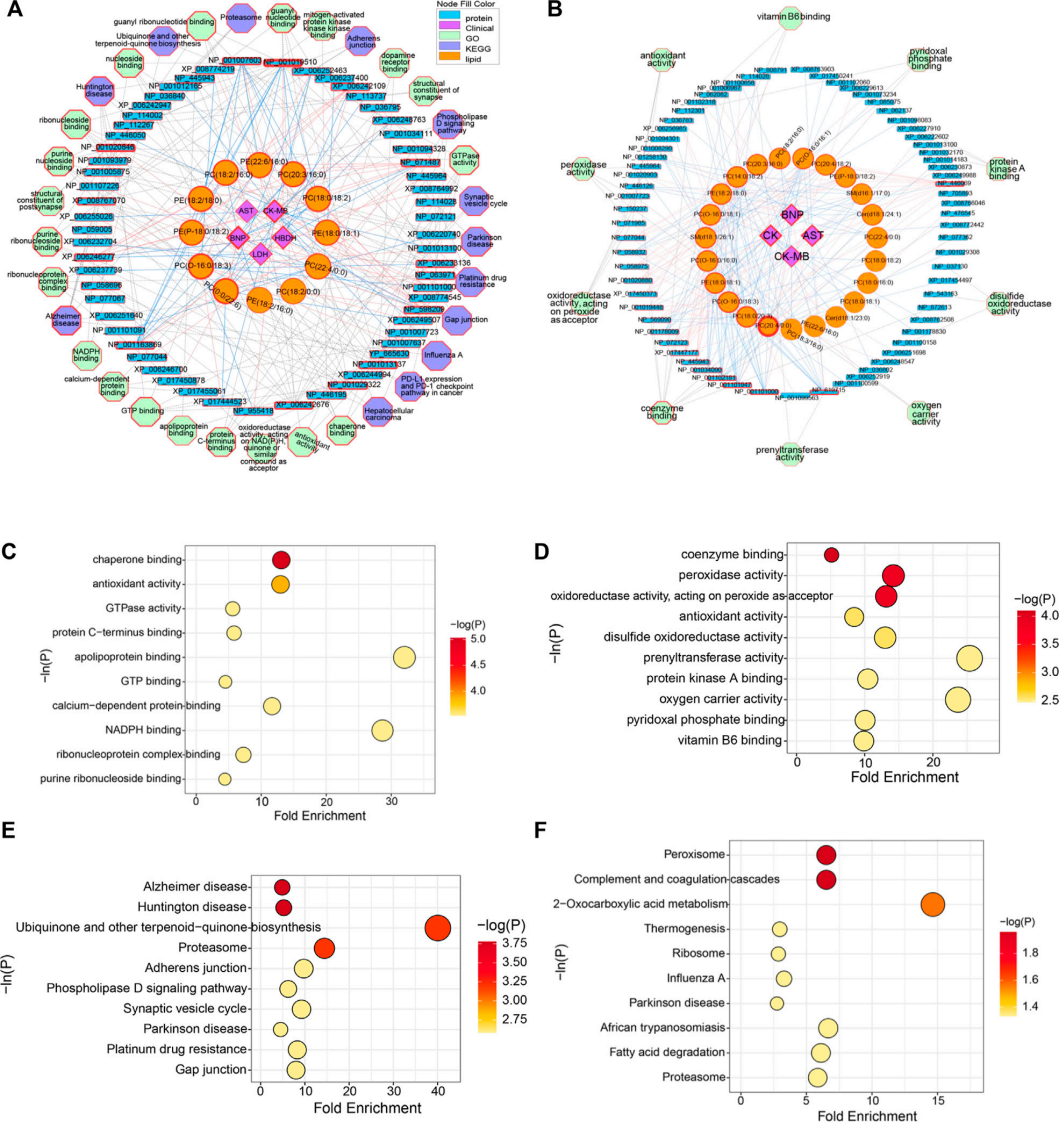
**(A)** Multi-layered network analysis based on differential lipids, differential proteins, CFA indexes and metabolic pathways in low-lose ArBu group. **(B)** Multi-layered network analysis based on differential lipids, differential proteins, CFA indexes and metabolic pathways in high-lose ArBu group. **(C)** Bubble chart of the first ten pathways of Go enrichment analysis based on low-dose ArBu group and control group. **(D)** Bubble chart of the first ten pathways of KEGG enrichment analysis based on low-dose ArBu group and control group. **(E)** Bubble chart of the first ten pathways of GO enrichment analysis based on high-dose ArBu group and control group. **(F)** Bubble chart of the first ten pathways of KEGG enrichment analysis based on high-dose ArBu group and control group.

Based on the above analysis, differential lipids, differential proteins and CFA indexes were correlated, and further related to the metabolic pathways involved (Figures 6A,B). It could be seen from the low-dose multi-layered network that BNP, CK-MB and HBDH were important cardiotoxicity index. The main changes in lipids were PC(0:0/22:6), PC(O-16:0/18:3), PE(P-18:0/18:2), PE(18:2/18:0), PC(18:2/16:0), PE(22:6/16:0), PC(20:3/16:0), PC(18:0/18:2), PE(18:0/18:1) and PC(22:4/0:0). The key proteins were NP_001019510 (Tuba8), NP_671,487 (Rwdd1), XP_006233136 (Rhoc), NP_598,209 (Dnph1), NP_001013137 (Gna13), NP_001029322 (Srpra), XP_006242676 (Dnm2), NP_001163869 (Fry), XP_006246277 (Sqstm1), XP_008767070 (Mapk1) and NP_001020846 (Tubb6). The main pathways involved in the molecular mechanism of low-dose ArBu were chaperone binding, Alzheimer disease and Huntington disease (Figures 6A,C,D). Significantly differential lipids in the high-dose ArBu group included PC(20:4/0:0) PC(20:4/0:0), PC(18:0/20:3), PC(O-16:0/18:3), PE(18:0/18:1), PC(O-16:0/16:0), SM(d18:1/26:1), PC(O-16:0/18:1), PE(18:2/18:0), PC(14:0/18:2), PC(20:3/16:0), PC(18:2/16:0), PC(O-16:0/16:1), PC(20:4/18:2), PE(P-18:0/18:2), SM(d16:1/17:0), Cer(d18:1/24:1) and PC(22:4/0:0). Important nodal proteins played key roles in exploring the mechanism of cardiotoxicity. These proteins were mainly NP_446,069 (Cpb2), NP_619,715 (Rabggtb), NP_001101000 (Arpin), NP_001101947 (Hnrnpul1), NP_001102181 (Snx6), NP_001034090 (Trap1), NP_445,943 (Plg), XP_017447177 (Xirp2) and NP_001178009 (Golga4). Combining the multi-layered network and bubble diagram (Figures 6B,E,F), the metabolic pathways involved in the molecular mechanism of high-dose ArBu were mainly coenzyme binding, oxidoreductase activity (acting on peroxide as acceptor), peroxidase activity, and complement and coagulation cascades.

### 3.6 Protein Verification by LC-MS/MS in PRM Mode

In view of proteomics data analysis results, the relationships and status in the above two network diagrams, biological implications and references, important differential proteins were selected for protein validation using PRM mode. In PRM technology, all ions produced were detected by a high-resolution detector following the fragmentation of the precursor ions. This was the difference between the PRM and MRM. Due to the different detection modes of secondary MS, PRM technology was more accurate than MRM technology in terms of qualitative and quantitative accuracy.

**FIGURE 7:**
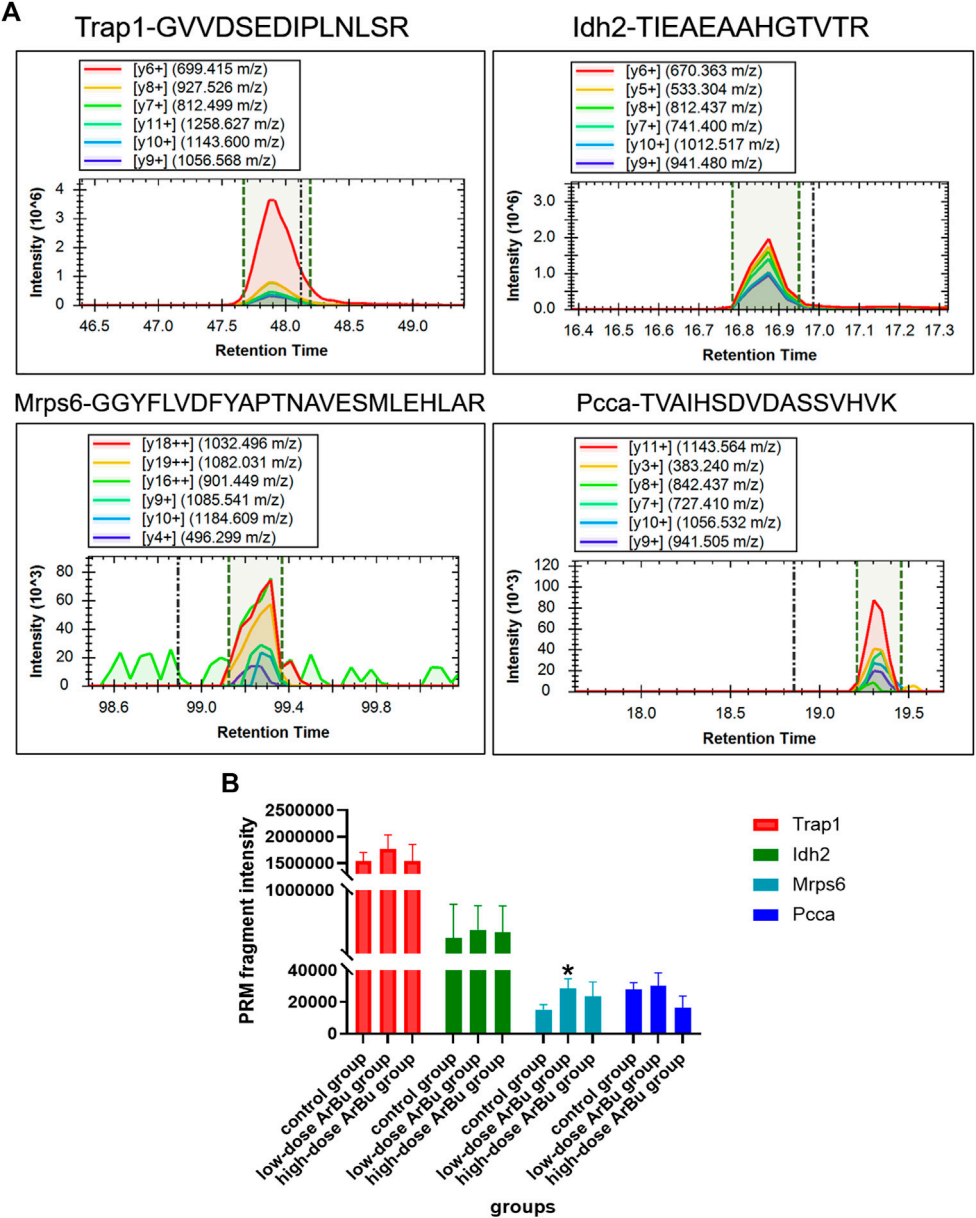
Protein verification by a targeted PRM assay. **(A)** The fragment ions with highest intensities in the characteristic peptides of Trap1, Idh2, Mrps6 and Pcca were used for PRM quantification. **(B)** Relative abundance of Trap1, Idh2, Mrps6 and Pcca in control, low- and high-dose ArBu groups. The data were expressed as mean value ±SD. *means differential proteins in the administration groups were significantly different from that in control group (*p < 0.05).

After screening, 23 differential proteins were used as verification targets, including Idh2, Fermt3, S100A1, Mrps6, mrpl9, Plg, Trap1, Prdx3, CPB2, Snx6, Golga4, Xirp2, CMA1, Ddx17, Atp8b2, Dnm2, Cbr4, Nebl, Mrpl16, F13a1, Popdc2, TMEM16F and Pcca. In order to ensure the reliability of the verification results, the differential protein with more than two characteristic peptides was determined as the further verification object. The abundance of proteins of interest was confirmed by targeted proteomics using PRM analysis. The proteomics results showed that mitochondrial proteins (Trap1, Idh2, Mrps6, Pcca), cell cycle-related proteins (Trap1), heart disease related proteins (Trap1, Mrps6), and important network node proteins (Trap1, Mrps6) were enriched in administration groups (Figure 7A). In the PRM verification, the regulation trends of ArBu on the four proteins were consistent with proteomics, but the number of characteristic peptides successfully verified for each protein was different (two for Trap1, three for Idh2, one for Mrps6, two for Pcca). In the low-dose ArBu administration group, Trap1 (GVVDSEDIPLNLSR), Idh2 (TIEAEAAHGTVTR), Mrps6 (GGYFLVDFYAPTNAVESMLEHLAR) and Pcca (TVAIHSDVDASSVHVK) were all highly expressed when compared with the control group. Among them, Mrps6 was statistically different from that in the control group. However, in the high-dose ArBu administration group, only Idh2 (TIEAEAAHGTVTR) and Mrps6 (GGYFLVDFYAPTNAVESMLEHLAR) were highly expressed (Figure 7B).

Tumor necrosis factor receptor-associated protein 1 (TRAP1), a molecular chaperone, was a major member of the mitochondrial heat shock protein 90 family. It was closely related to mitochondrial function and cell cycle. Mitochondrial dynamics played vital roles in calcium homeostasis and cell cycle regulation (Horbay and Bilyy, 2016; Kowaltowski et al., 2019). The alterations in mitochondrial dynamics were associated with cardiovascular diseases (Carreira et al., 2011; Dorn and Gerald, 2016; Ong and Hausenloy, 2017). TRAP1, an energy metabolism regulator, could prevent hypoxiainduced damage to cardiomyocytes, maintained cardiomyocytes viability and mitochondrial membrane potential, and protected from ischemic damage in cardiomyocytes (Voloboueva et al., 2008; Xiang et al., 2010; Li et al., 2020). TRAP1 overexpression promoted mitochondrial fission and dysfunction, protected cells against oxidative stress, maintained mitochondrial integrity, and triggered the cellular metabolism (Dharaskar et al., 2020; Ramkumar et al., 2020). In addition, TRAP1 could regulated the cell cycle and was closely related to mitochondrial autophagy (Liu et al., 2010; Caino et al., 2013; Costa et al., 2013; Siegelin, 2013; Chen et al., 2017).

Idh2 was known as Isocitrate dehydrogenase [NADP], mitochondrial. In the lipid-protein network map (high dose), Idh2 was closely related to coenzyme binding. It was closely associated with mitochondrial dysfunction. Reactive oxygen species induced oxidative stress was an important component of cardiovascular diseases such as hypertension, cardiac hypertrophy and atherosclerosis (Madamanchi and Runge, 2007; Doughan et al., 2008; Dikalova et al., 2010; Dai et al., 2011). Idh2 was induced by reactive oxygen species, which might be attributed to increased levels of Idh2 in ArBu administration groups. Idh2 was a major NADPH producer in the mitochondria and played a critical role in cellular defense against oxidative stress-induced damage (Madamanchi and Runge, 2007).

The full name of Mrps6 was mitochondrial ribosomal protein S6, which was an important nodal protein in PPI. Mrps6 showed an increasing trend in mitochondrial diseases, cardiovascular diseases, myocardial infarction and coronary artery diseases. In addition, it was involved in cell apoptosis, signal transduction and cell cycle. MRPS6 encoded a subunit of the mitochondrial ribosome (Suzuki et al., 2001). It was an unique and essential component for the structural and functional integrity of the mitoribosome complex (Cheong et al., 2020). The increased expression of the risk allele for coronary heart disease was closely related to the expression of MRPS6 (Beaney et al., 2017). Cardiomyopathy as a kind of mitochondrial disorder was related to mutations in Mrp genes (De Silva et al., 2015). Mrp genes were also involved in cell proliferation and apoptosis (Wu et al., 2019; Klæstad et al., 2020).

The full name of Pcca was propionyl-CoA carboxylase alpha chain, mitochondrial. Pcca was associated with genetic metabolic disorders and calcium metabolism. Pcca was closely related to metabolic diseases, and cardiac dysfunction was one of its important manifestations (Massoud and Leonard, 1993; Baumgartner et al., 2014; Richard et al., 2015). There existed a strong relationship between cardiac dysfunction, arrhythmia and the changes in intracellular Ca^2+^ homeostasis. The increased expression of Pcca in the ArBu administration groups affected the impairment of systolic Ca^2+^ release and to irregular diastolic Ca^2+^ release, leading to cardiac and cellular contractility abnormalities. These changes were also associated with increased levels of oxidative stress in cardiomyocytes (Tamayo et al., 2020). In conclusion, Trap1, Pcca, Mrps6 and Idh2 were potential biomarkers for the cardiotoxicity of ArBu.

### 3.7 Cardiotoxicity of ArBu

Based on the changes of heart rates, the increase of myocardial enzyme and BNP levels, the disorder and fracture of myocardial fibers in pathological sections, ArBu plasma concentration-time profiles of toxicokinetics, differential lipids and differential proteins, it could be concluded that ArBu had cardiotoxicity. However, ArBu was a very good broad-spectrum anti-tumor candidate compound, so it was parsticularly significant to study and overcome the cardiotoxicity of ArBu.

Some measures could be taken to reduce the cardiotoxicity of ArBu. Improving the dosage form could reduce the cardiotoxicity of bufogenins. It has been reported that the distribution of bufadienolides mixture (bufalin: cinobufagin: bufadienolide, 2: 3: 5) in the heart could be decreased by nanostructured lipid carrier drug delivery (Li et al., 2010). Albumin nanoparticles reduced the bufalin concentration in the blood to decrease its toxicity (Zhang et al., 2012). Additionally, changing the mode of administration could avoid cardiotoxicity, for example, topical administration. Moreover, previous studies have demonstrated that combined administration decreased the cardiotoxicity of bufadienolide in guinea pigs or mice, such as taurine, bilirubin, bezoar treasure and ouabain (Nesher et al., 2010; Ma et al., 2012a; Ma et al. 2012b; Ma et al. 2012c).

## 4 Conclusion

In the present investigation, a verified method was successfully applied to characterize the toxicokinetics of ArBu in rats. The cardiotoxicity mechanism of ArBu was studied by lipidomics and proteomics using the LC-MS/MS technique. Toxicity studies demonstrated that heart rates varied with the dosage of ArBu. Low-dose ArBu accelerated heart rates. However, the heart rates increased slightly and then decreased after high-dose ArBu treatment. Over a period of time, they all went back to normal, which was in line with the trend of ArBu concentration-time curve. Increased myocardial enzymes and BNP indicated that ArBu inhibited or impaired cardiac function. Pathological sections of livers showed that ArBu had no hepatotoxicity. However, the pathological sections of hearts in high-dose (more serious) and low-dose ArBu groups showed certain cardiotoxicity (muscle fiber disorder or rupture). Lipidomics analysis revealed the difference between serum and heart differential lipids. The difference was positively correlated with the dosage. Furthermore, cardiotoxicity was related to a variety of CFA indexes. The cardiotoxicity of ArBu was also reflected in proteomics research. Proteomics demonstrated that the differential proteins affected the calcium ion levels, mitochondrial energy metabolism, blood coagulation, lipid metabolism and heart-related diseases. Mrpl16, Mrps6, Prdx3, F13a1, mrpl9 and Ddx17 were significant nodal proteins in the PPI diagram. The multi-layer network analysis revealed the difference of the pathways involved in different ArBu doses. In the low-dose ArBu group, they were mainly chaperone binding, Alzheimer disease and Huntington disease. However, in high-dose ArBu group, these were coenzyme binding, oxidoreductase activity (acting on peroxide as acceptor), peroxidase activity, and complement and coagulation cascades. PRM verification showed that the regulation trends of ArBu on Trap1 (low-dose group), Idh2 (low- and high-dose group), Mrps6 (low- and high-dose group) and Pcca (low-dose group) were consistent with proteomics. Trap1, Idh2, Mrps6 and Pcca were potential biomarkers for the cardiotoxicity of ArBu. In conclusion, high-dose ArBu caused inhibition or damage of cardiac function in rats. From cardiac excitation to cardiac inhibition, the underlying molecular mechanisms were quite different. These results provided a theoretical basis for a comprehensive regulation of the microenvironment on the cardiotoxicity effects of ArBu.

## Data Availability

The datasets presented in this study can be found in online repositories. The names of the repository/repositories and accession number(s) can be found below: http://www.proteomexchange.org/, PXD028622.

## Glossary

ArBu: arenobufagin
AST: aspartate aminotransferase
AUC: area under curve
BNP: brain natriuretic peptide
CFA: cardiac function assessment
CE: cholesteryl ester
Cer: ceramide
CK: creatine kinase
CK-MB: creatine kinase isoenzyme
CL: cardiolipins
Cmax: maximum observed concentration of drug in plasma
DG: diacyl glycerol
FCmax: maximum fold change
FCmin: minimum fold change
FOT: a fraction of total protein iBAQ amount per experiment
GO: gene ontology consortium
HBDH: hydroxybutyrate dehydrogenase
iBAQ: intensity-based-absolute-protein-quantification
LC-MS/MS: liquid chromatography-tandem mass spectrometry
LDH: lactate dehydrogenase
LQC: low concentration quality control
Lyso-PA: lyso-phosphatic acid
KEGG: kyoto encyclopedia of genes and genomes
MQC: medium concentration quality control
MRM: multiple reaction monitoring mode
OPLS-DA: orthogonal projection to latent structures discriminate analysis
PRM: parallel reaction monitoring
PC: phosphatidylcholine
PE: phosphatidylethanolamine
PS: phosphatidylserine
PG: phosphatidylglycerol
PCA: principal components analysis
PPI: protein–protein interaction
RSD: relative standard deviation
SM: sphingomyelin
TG: triglyceride
Tmax: time of Cmax
UniProt: universal protein
VIP: variable influence on the projection
