# Supplementary material for: Toxicokinetics of Arenobufagin and its Cardiotoxicity Mechanism Exploration Based on Lipidomics and Proteomics Approaches in Rats
Source: Front Pharmacol. 2021 Dec 22;12:780016. doi: 10.3389/fphar.2021.780016 (PMC8727535; doi:10.3389/fphar.2021.780016)
Supplement: Supplementary file 1 [file DataSheet1.DOC]

Supplementary Material

# Supplementary Figures


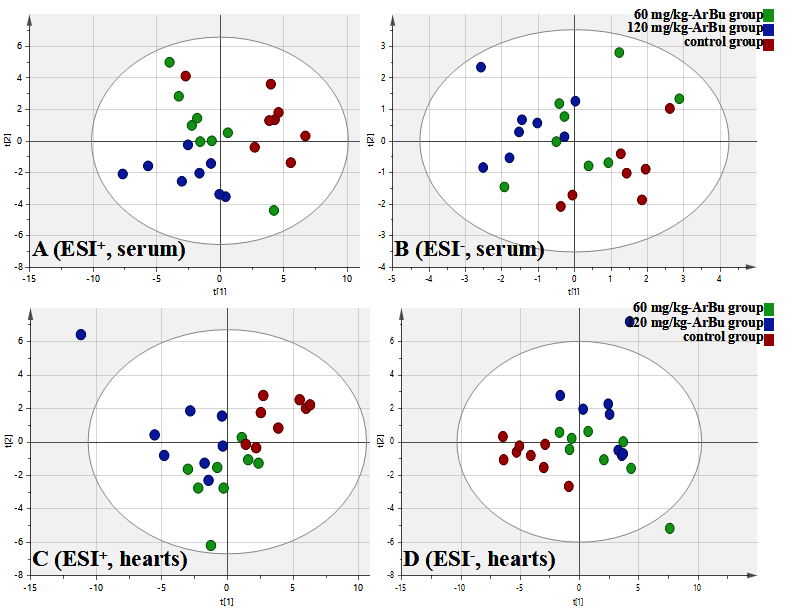


**Supplementary Figure 1.** The ESI^+^ and ESI^-^ of metabolic profiles PCA charts obtained from serum and hearts.


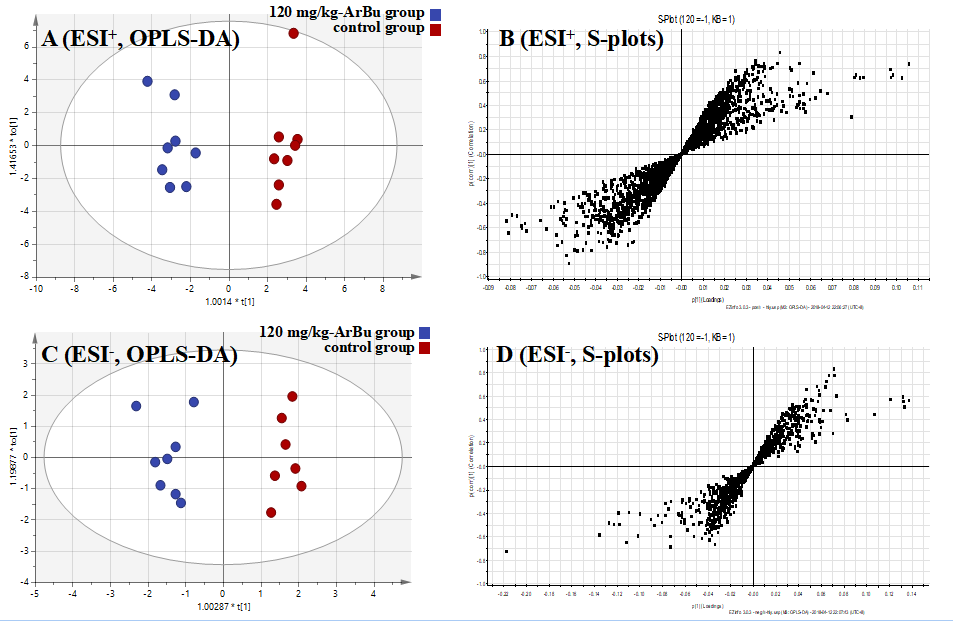


**Supplementary Figure 2.** The ESI^+^ and ESI^-^ of metabolic profiles OPLS-DA charts and *S*-plot charts obtained from serum.


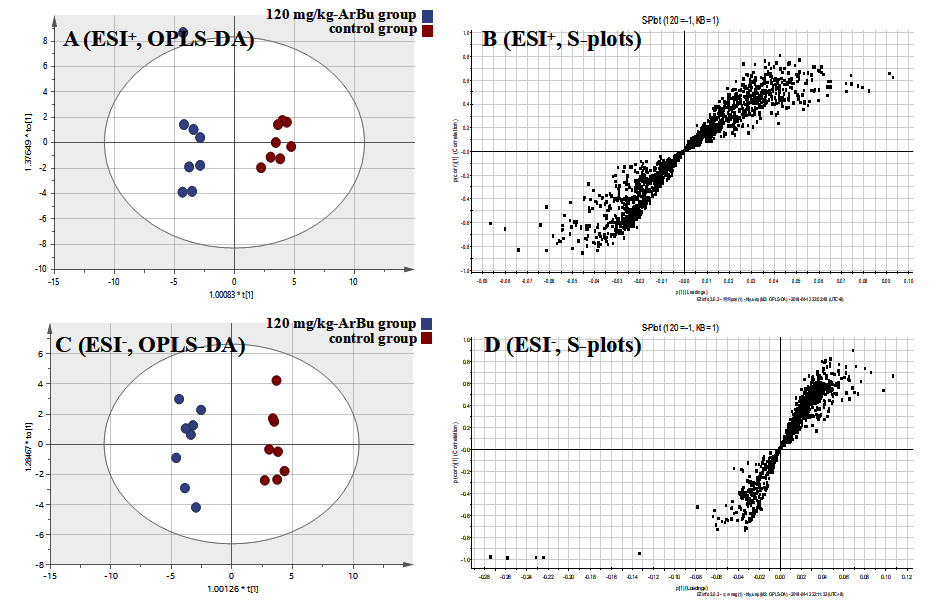


**Supplementary Figure 3.** The ESI^+^ and ESI^-^ of metabolic profiles OPLS-DA charts and S-plot charts obtained from hearts.


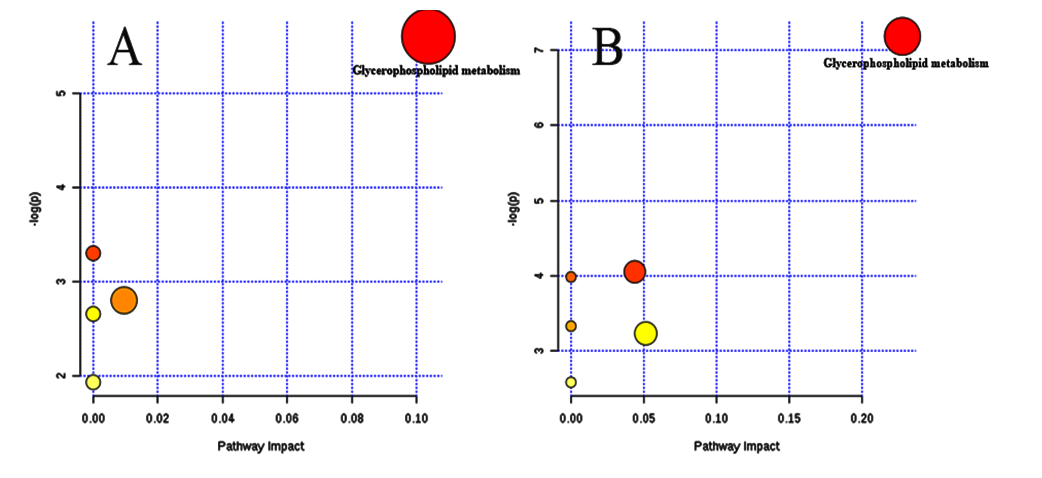


**Supplementary Figure 4.** Metabolic pathway analysis of differentially-expressed lipids. A, serum; B, hearts.
